# Genome-wide identification and integrated analysis of gibberellin oxidase genes associated with branch angle and internode elongation in tea plants (*Camellia sinensis*)

**DOI:** 10.3389/fpls.2026.1940210

**Published:** 2026-09-03

**Authors:** Hong Shen, Liping Zhang, Hanjia Li, Shibei Ge, Haoyang Liu, Yang Li, Peiqiang Wang, Jinping Zhu, Ahmed S. Mohamed, Xin Li, Jianming Zeng, Shuixing Zhu

**Affiliations:** 1 State Key Laboratory of Tea Plant Germplasm Innovation and Resource Utilization, Tea Research Institute, Chinese Academy of Agricultural Sciences, Hangzhou, China; 2 College of Horticulture, Qingdao Agricultural University, Qingdao, China; 3 Horticultural Crops Technology Department, Agricultural and Biological Research Institute, National Research Centre, Giza, Egypt; 4 Agricultural Technology Promotion Center in Xianju County, Taizhou, China

**Keywords:** branch angle, gene family, gene pair, gibberellin, internode length, mechanical picking, new shoot, tea

## Abstract

**Introduction:**

Gibberellin oxidases (GAoxs) are key enzymes involved in maintaining gibberellin (GA) homeostasis and therefore play important roles in regulating plant architecture. However, the GAox genes associated with branch angle and internode elongation in tea plants (*Camellia sinensis*) remain largely uncharacterized.

**Methods:**

In this study, 33 *CsGAox* genes were identified in the genome of the tea cultivar `Longjing 43' and classified into four subfamilies. Comparative genomic analysis across eight Camellia genomes was performed, and phenotypic measurements were integrated with endogenous GA quantification, transcriptome profiling, qRT-PCR analysis, and exogenous GA₃ treatment.

**Results:**

Whole-genome or segmental duplication contributed substantially to the expansion of the *CsGAox* family, with duplicated gene pairs in `Longjing 43' predominantly undergoing purifying selection. The levels of GA₁, GA₉, and GA₂₀ were higher in the lateral buds of the draped cultivar than in those of the upright cultivars and were positively correlated with branch angle. *CsGA20ox4.3* expression was positively associated with branch angle and the levels of those GAs, whereas *CsGA2ox5.3* exhibited the opposite relationships. Exogenous GA₃ application significantly increased branch angle and was accompanied by the upregulation of *CsGA20ox4.3* and *CsGA20ox.un3* and the downregulation of *CsGA2ox5.3*. Integrated analyses across cultivars and developmental stages further showed that *CsGA20ox4.3* and *CsGA3ox3.3* were positively associated with internode elongation, whereas*CsGA2ox1.1*, *CsGA2ox5.2*, and *CsGA2ox.un1* generally exhibited negative associations.

**Discussion:**

These findings identify candidate *CsGAox* genes associated with branch angle and internode elongation, providing a basis for future functional studies and further evaluation in tea plant architecture breeding.

## 1 Introduction

The tea plant (*Camellia sinensis*) is a perennial woody cash crop cultivated worldwide, and the manual harvesting of fresh tea leaves is the most labor-intensive work in tea production (Sun et al., 2018). With the increasingly severe problems of high labor costs and labor shortages, mechanical picking is an inevitable option for the tea industry in the future (Luo et al., 2023). Tree architecture is vital for tea mechanical harvesting. The branch angle and internode length in tea new shoots are two key traits that determine the suitability of tea plants for mechanical picking. However, the related genes and molecular mechanisms regulating tree architecture remain largely unknown.

Plant hormone gibberellins (GAs) play major roles in regulating plant branch angle and internode length (Liu et al., 2024; Dutt et al., 2022; Li Y. et al., 2021; Honi et al., 2020). For example, *OsGRF7* overexpression causes a narrower tiller angle through regulating the expression of the GA-biosynthetic gene *cytochrome P450* in rice (Zhu et al., 2025). Modification of GA signaling via DELLA proteins produces a narrower branch angle. *TAC1*-edited plants increased the branch angle through increasing GA_7_ level in citrus leaves (Dutt et al., 2022). On the other hand, exogenous GA application promoted internode elongation of tea plants (Li W. et al., 2021), jute (Honi et al., 2020), and deep-water rice (Davière et al., 2014). Endogenous GA_3_ levels significantly increase during new shoot elongation (Li W. et al., 2021) and are strongly associated with internode length of tea plants (Luo et al., 2023). Mutants defective in GA biosynthesis or perception typically have shorter stems (Davière et al., 2014). Compared with the tall cultivar, the decreased expression of GA biosynthetic genes resulted in a greater decrease in the accumulation of active GA_1_ in the dwarf cultivar of *C. purpureus* (Yan et al., 2021). Similarly, the tea cultivars with longer internodes presented higher levels of GA_1_ and GA_3_, and a highly significant correlation was observed between GA_3_ levels and internode length (Luo et al., 2023).

Bioactive GAs are signaling molecules essential for plant growth and development (Lange et al., 2020). The major bioactive GAs are GA_1_, GA_3_, GA_4_, and GA_7_, while the others are the precursors or deactivated forms of bioactive GAs (Luo et al., 2023). Gibberellin homeostasis is achieved via complex biosynthetic and catabolic pathways (Lange et al., 2020). Gibberellin oxidase (GAox) enzymes, including GA 20-oxidase (GA20ox), GA 3-oxidase (GA3ox), and GA 2-oxidase (GA2ox), belong to the 2-oxoglutarate-dependent dioxygenases (2-ODDs) family and catalyze the last steps of the GA metabolic pathway in higher plants (Pan et al., 2017; Hu et al., 2021; Lange et al., 2020). GA20ox and GA3ox enzymes are anabolic enzymes of bioactive GAs (Lange et al., 2020; He et al., 2019; Sun et al., 2023), whereas GA2oxs are catabolic enzymes (Li Y. et al., 2021; Hu et al., 2021; Lange et al., 2020; Ren et al., 2024). Specifically, GA20ox enzymes catalyze successive oxidation at C-20, converting inactive GA_12_ and GA_53_ into GA_9_ and GA_20_, respectively (Lange et al., 2020; He et al., 2019; Sun et al., 2023). In the final step of bioactive GA formation, GA3ox enzymes catalyze the 3*β*-hydroxylation of GA_20_ and GA_9_ to produce bioactive GA_1_ and GA_4_, respectively (Hu et al., 2021; He et al., 2019; Plackett et al., 2012; Song et al., 2011). GA2ox enzymes include two major groups, C_19_-GA2ox and C_20_-GA2ox, which act on C_19_-GAs and C_20_-GAs, respectively (Plackett et al., 2012). Through 2*β*-hydroxylation, C_19_-GA2ox enzymes inactivate C_19_-GAs (GA_1_ and GA_4_) as well as their immediate precursors (GA_20_ and GA_9_), producing inactive GA_34_, GA_8,_ GA_51_, and GA_29_, while C_20_-GA2ox enzymes oxidize C_20_-GAs (GA_12_ and GA_53_) to inactive GA_110_ and GA_97_ (Pan et al., 2017; Plackett et al., 2012; Song et al., 2011).

Previous studies showed that GAoxs regulate plant angle at the transcriptional level. For example, the asymmetric expression of *OsGA3ox1* regulated the gravitropic growth of rice leaf sheath through triggering cell wall loosening (Zhu et al., 2025). GA2ox overexpression increased the branch angle by reducing the levels of bioactive GAs (Dutt et al., 2022). On the other hand, GAoxs also play key regulatory roles in plant internode elongation. For example, *AtGA20ox1* and *AtGA20ox2* act redundantly to promote internode elongation (Fukazawa et al., 2017). Mutations in *GA20ox* or *GA3ox* genes generated dwarf or semi-dwarf phenotypes in *Arabidopsis*, *Zea mays*, and rice (Sun et al., 2023; He et al., 2025). The *ga20ox1*, ga*20ox2*, and ga*20ox3* mutants all showed a severe dwarfing phenotype (Plackett et al., 2012). Overexpression of *GA2ox* genes results in a dwarf phenotype in *Arabidopsis*, Jatropha, and *C. lipoensis* (Honi et al., 2020; He et al., 2019; Ren et al., 2024), while silencing of the *GA2ox* gene in transgenic tobacco leads to increased plant height and xylem cell number (Honi et al., 2020).

After the release of the genome of tea cultivar Yunkang10 (YK10), Pan et al. performed a genome-wide study of the CsGAox gene family in YK10 (Pan et al., 2017). At present, multiple Camellia genomes have been released, including those of the tea cultivars Biyun (BY), Huangdan (HD), Shuchazao (SCZ), Tieguanyin (TGY), LJ43, Yunkang10 (YK10), the related species C. oleifera (COL), C. chekiangoleosa (CCH), and the wild tea plant DASZ. Given the important roles of GAox genes in GA metabolism and plant development, a genome-wide survey of the *CsGAox* gene family was conducted using eight Camellia genomes. The bioinformatic characteristics of CsGAox genes in LJ43 and their associations with branch angle and internode length in machine-picked tea plants were investigated. Furthermore, to gain insights into the evolutionary diversity of *CsGAox* genes, the syntenic relationships and gene duplication patterns between LJ43 and the other seven genomes were comparatively analyzed.

In this study, 33 *CsGAox* genes were identified from the genome of tea cv. ‘LJ43’ and classified into four subfamilies based on phylogenetic analysis, gene structure, and conserved motifs. Synteny analysis indicated that whole-genome duplication (WGD) or segmental duplication (SD) events played major roles in the expansion of *CsGAox* members. Comparative syntenic analysis combined with phylogenetic analysis provided deep insight into the phylogenetic relationships of *CsGAox* genes. Integrated analyses of phenotypes, GA contents, and gene expression levels indicated *CsGA20ox4.3*, *CsGA20ox.un3*, and *CsGA2ox5.3* as candidate genes associated with branch angle, while *CsGA20ox4.3*, *CsGA3ox3.3*, *CsGA2ox1.1*, *CsGA2ox5.2*, and *CsGA2ox*.*un1* were identified as candidate genes associated with internode elongation in tea plants. The expression responses of *CsGAox* genes to exogenous GA_3_ treatment further supported the association of the above three candidate genes with GA-responsive branch angle formation. Meanwhile, exogenous GA_3_ treatment altered the expression of several *CsGAox* genes, consistent with transcriptional feedback responses in GA metabolism. Taken together, this study identified candidate *CsGAox* genes associated with tea plant architecture, providing candidates for future functional studies and further evaluation in tea plant architecture breeding for mechanical harvesting.

## 2 Materials and methods

### 2.1 Identification of GAox family members in tea plants

To identify *CsGAox* family genes in the tea plant genome, a BLASTP search was performed using the sequences of 16 *Arabidopsis* AtGAox proteins as queries against eight Camellia genome databases (e-value ≤ 1×10^-5^) (https://tpia.teaplants.cn/index.html#). Furthermore, the Hidden Markov Model (HMM) profiles of the 2OG-FeII_Oxy (PF03171) and DIOX_N (PF14226) domains were obtained from the Pfam database (http://pfam.janelia.org). Candidate proteins containing both domains were identified using HMMER v.3.2 with an E-value cutoff of 1 × 10−10 (Sun et al., 2018; Ren et al., 2024). The putative *CsGAox* genes were further validated by the NCBI Conserved Domain Database (CDD) and online Simple Modular Architecture Research Tool (SMART) (http://smart.embl.de). All of the 33 putative *CsGAox* genes from tea cv. LJ43 were shown in **Supplementary Table 1**; the sequences of the 33 CsGAox and 16 *Arabidopsis* AtGAox proteins were listed in **Supplementary Table 2**. For the eight Camellia genomes, the numbers of identified *CsGAox* genes were listed in **Supplementary Table 3**.

### 2.2 Physicochemical property analysis of LJ43 CsGAox proteins

The physicochemical properties, including amino acid (AA) number, isoelectric point (pI), molecular weight (MW), grand average of hydropathicity (GRAVY), instability index, and aliphatic index, were predicted using the ExPASy website tool (https://www.expasy.org/). The subcellular localization was predicted using DeepLoc-2.0.

### 2.3 Multiple sequence alignment, phylogenetic analysis, conserved motif analysis, and gene structure analysis of LJ43 CsGAox proteins

The amino acid sequences of 33 putative CsGAox proteins from LJ43 and 16 AtGAox proteins from *Arabidopsis thaliana* were aligned using MUSCLE implemented in TBtools. The multiple sequence alignment was visualized using Jalview. A maximum-likelihood (ML) phylogenetic tree was constructed using MEGA 7.0 under the Poisson amino acid substitution model with 1,000 bootstrap replicates. No separate model-selection procedure was performed. The resulting phylogenetic tree was visualized using FigTree. The conserved motifs were predicted using the MEME online software (http://meme-suite.org/tools/meme) (maximum motif = 10, width = 6–50). The gene structures (exon and intron) were analyzed and visualized using the online tool Gene Structure Display Server (GSDS v.2.0, http://gsds.cbi.pku.edu.cn/). The gene structure information of 27 LJ43 *CsGAox* genes located on chromosomes was also listed in **Supplementary Table 3**.

### 2.4 Chromosome mapping, gene duplication analysis, and selective pressure analysis

The chromosomal location maps of *CsGAox* genes were constructed based on the genome annotation file of eight Camellia genomes using TBtools. The duplication events of *CsGAox* genes, including the gene collinearity relationships, were analyzed using MCScanX (https://chibba.agtec.uga.edu/mcscanx-web) and visualized with TBtools. Tandem duplication events were determined according to the following two conditions: (1) the relatively constant short sequences that are more than 70% of the length of the long sequences; (2) the similarity of two aligned sequences is greater than 70%. Tandemly duplicated genes were defined as homologous genes located within a 100-kb region on the same chromosome and separated by fewer than five intervening genes (Jiao et al., 2024). The synonymous *(Ks*) and non-synonymous (*Ka*) values as well as the *Ka/Ks* ratios for each gene pair were calculated using the KaKs Calculator 2.0 (https://sourceforge.net/projects/kakscalculator2/).

### 2.5 *Cis*-acting element analysis

The promoter sequences (1,000 bp) of 33 *CsGAox* genes in LJ43 were extracted using TBtools, and the promoter elements were predicted and analyzed using the PlantCARE online tool (http://bioinformatics.psb.ugent.be/).

### 2.6 Gene expression analysis of CsGAox genes associated with tea plant architecture

To investigate the associations between *CsGAox* gene expression and tea plant architecture, we analyzed their FPKM values using transcriptome datasets from our research group and a publicly available dataset. Our own RNA-seq results were further verified by quantitative real-time PCR (qRT-PCR).

### 2.7 Gene expression analysis of CsGAox genes associated with branch angle formation in tea plants

Two upright tea cultivars (*C. sinensis* L.), CT2009-0025 (upright tea 1, UT1) and CT2013-0114 (UT2), and one draped tea cultivar, CT2009-0160 (DT), were selected as experimental materials. The draped cv. DT was defined as the control. The branch angle of the three cultivars can be seen in **Supplementary Figure 2**, and the cultivation conditions, decapitation treatment, sampling, and measurement of branch angles were shown in Section 4.1 of our recent study. The lateral buds were sampled for transcriptome sequencing, qRT-PCR testing, and hormone measurement (Zhu et al., 2025). As shown in Section 4.6 of the recent study, exogenous GA_3_ treatment was conducted using the upright tea cv. Zhongcha 108 (ZC108). The cultivation conditions and pruning treatment were described in the same study (Zhu et al., 2025). GA_3_ solutions at concentrations of 0.5, 5, 50, and 100 mg/L were sprayed onto the tea plants, with an equal volume of pure water serving as the control. On the 20th day after GA_3_ spraying, the branch angle between the shoot stem and the first lateral bud was measured. The lateral buds were sampled for hormone measurement and qRT-PCR testing (Zhu et al., 2025).

Here, the FPKM values of *CsGAox* genes were analyzed based on these transcriptome data. Total RNA was extracted using the CTAB method. The extracted RNA was treated with DNase I (Invitrogen, CA, USA). The integrity, concentration, and purity of all the RNA samples were analyzed and monitored. The reverse transcription was carried out using a PrimeScript™ RT-PCR Kit (RR047A, TaKaRa, China), and cDNA was diluted 10-fold. The gene-specific primers were designed using Primer 5.0, and the primer sequences and Tm were listed in **Table 1**. *CsGAPDH* was selected as the reference gene. The qRT-PCR assay was carried out using an ABI 7500 Real-Time PCR System (Applied Biosystems, Foster City, CA, USA) with a SYBR Green I Master kit (Roche, Basel, Switzerland). Each reaction was performed in a total volume of 20 μL, with each forward and reverse primer at a final concentration of 0.2 μM. The thermal cycling program consisted of an initial denaturation at 95 °C for 30 s, followed by 40 cycles of 95 °C for 5 s and 60 °C for 30 s for annealing and extension. Three independent biological replicates were analyzed for each sample. The relative expression levels of *CsGAox* genes were calculated using the 2^−ΔΔCT^ method.

**Table 1:** Primers of *CsGAox* genes used for qRT-PCR.

Gene names	Forward primer sequence (5′–3′)	Reverse primer sequence (5′–3′)	Tm (°C)
*CsGA20ox4.4*	GAGCCAATCGTCCGAGTCCA	CGGTCTCTGGTGGCGATTCT	59.0, 59.0
*CsGA2ox4.1*	GGGTCGAGAACTGTCGGACT	GCTCGGTTCTCCTCCGAACA	58.3, 58.9
*CsGA2ox5.3*	CCGGAGTTGATGGCGGAGAT	GAGCACCAGGGACACAGGAG	59.1, 59.2
*CsGA3ox15*	CTCGATCGCCAGAGGGTGTT	GGGCCAAAGTTGGCAAGCAT	59.0, 59.1
*CsGA20ox4.3*	ACCGAAGCCTGTGAGACCTG	CGACTCGATTGCTCGGCTCT	58.9, 59.5
*CsGA2ox7.3*	TGAAGGTGTTCAAGAGGCCGTT	CAAGTCGCCGACGGTGTTC	59.1, 59.0
*CsGA20ox.un3*	GCAAAGAACCTGGCCTCACC	TCGAGGCCTCCAACTTCGTC	58.6, 58.9
*CsGA2ox1.1*	CCGAGTCGACCGAGTTGTCA	GTTACCCGGAGACCCTTCCG	59.0, 59.0
*CsGA2ox5.2*	CGATGCGAGCCAAGAATGGG	TACCCACTTCCGGCGATGAC	58.9, 59.0
*CsGA2ox5.4*	CCGACACGAAGACCCTCCTC	TGAGTTCTGTTGGGACGCCAT	59.0, 58.8
*CsGA2ox.un1*	AACCTGCAAGCTCACCACCA	AGTTGGGACGCCATGGTTGA	59.3, 59.1
*CsGA3ox3.3*	TAGCTGCTCACACGGACTCG	GGGTTGTGGAGGAACCGTGA	59.0, 59.1
*CsGAPDH*	GAGACTGGAGCCGAATTCATT	GATCTGGCTTGTAATCCTTCTCA	59.0, 59.0

### 2.8 Gene expression analysis of CsGAox genes associated with internode elongation in tea plants

Mature plants of two tea cultivars, Longjing 43 (LJ43) and Zhongcha 102 (ZC102), were used in this study. These two cultivars have significantly different internode lengths, and both were selectively bred from the Longjing population. Therefore, the two cultivars have a similar genetic background. The cv. LJ43, with a longer internode, was defined as the control. The tea plants were lightly pruned after spring tea picking. At the 2-, 3-, and 5-leaf stages, the internodes in the new shoots on the first node emerging just below the trimmed cuts were selected as research objects. The internode length of each node was measured, respectively, and the average internode length was calculated. The samples of the mixed internodes were collected for transcriptome sequencing and measurement of GA contents (Zhang et al., 2026). Here, the FPKM values of *CsGAox* genes were analyzed based on these transcriptome data. Total RNA extraction, reverse transcription, and qRT-PCR assay protocols were the same as described in Section 2.7.

In a previous report, transcriptome sequencing was conducted to study the regulatory mechanism of internode length in three tea cultivars, LJ43, Qiancha 1 (QC1), and Feiyun (FY), whose internode lengths increased successively. The cv. LJ43 was defined as the control. When the tea plants reached the stage of one bud and two leaves, the internode length was measured, and the stem tissues were sampled for transcriptome sequencing. Here, the raw data of the internode length in the three cultivars were downloaded from the previous study (Mi et al., 2025). The raw transcriptome data were downloaded from the National Genomics Data Center (https://ngdc.cncb.ac.cn, PRJCA036838) and analyzed. The reads in the downloaded transcriptome data were mapped back onto the sequences of representative transcripts in LJ43 using the ‘Kallisto Super GUI Wrapper’ Plugin in TBtools. The FPKM values of all the representative transcripts in LJ43 were obtained, and the FPKM values of *CsGAox* genes were extracted subsequently.

### 2.9 Determination of GA contents

The measurement of endogenous GA contents was carried out by Nanjing Ruiyuan Biotechnology Co., Ltd., China. Three independent biological replicates were analyzed for each cultivar or treatment. Briefly, 50 mg of fresh lateral buds and 1.0 g of fresh internode samples were collected for the branch-angle and internode-length experiments, respectively, and ground in liquid nitrogen. Fifty microliters of an internal-standard solution containing isotope-labeled GA standards and 1 mL of a water-acetonitrile solution containing 1% formic acid were added sequentially. The samples were vortex-mixed, extracted, and centrifuged. The supernatant (800 μL) was collected and dried under a stream of nitrogen gas. The dried extracts were reconstituted in 100 μL of water–acetonitrile (1:1, v/v), followed by centrifugation. GA contents were determined using an Agilent 1290 Infinity UHPLC system (Agilent Technologies) coupled to a 5500 QTRAP mass spectrometer (AB SCIEX). GA contents were quantified according to the validated UHPLC–MS/MS quantification procedure of Nanjing Ruiyuan Biotechnology Co., Ltd.

### 2.10 Statistical analysis

All data were analyzed using the SPSS 21.0 (SPSS, Inc., Chicago, IL, USA) software. Quantitative data were presented as mean ± SE. The significance of differences between control and treatment groups was assessed by Student’s *t*-test. Differences among the three branch-angle cultivars DT, UT1, and UT2, among all gene–treatment combinations in the exogenous GA_3_ experiment, among the six cultivar–developmental stage combinations in LJ43 and ZC102, and among the three tea cultivars LJ43, QC1, and FY were analyzed by one-way ANOVA followed by Duncan’s multiple range test. Differences were considered significant at *P* < 0.05.

## 3 Results

### 3.1 Genome-wide identification and bioinformatics analysis of *CsGAox* gene family in tea plants

To identify *CsGAox* family genes, a BLASTP search of nine Camellia genome databases was conducted using the sequences of *Arabidopsis* AtGAox proteins as queries (**Supplementary Table 2**). Because representative transcripts could not be extracted from the DASZ genome, *CsGAox* genes were not identified from DASZ. The *CsGAox* members were identified after manual screening, and the obtained protein sequences were further aligned to the Swiss-Prot database. Subsequently, the protein sequences containing ‘DIOX_N (PF14226) and 2OG-FeII_Oxy (PF03171)’ domains were screened from all the proteins of tea plants using the HMMER method. Based on the intersection of the BLASTP and HMMER search results, a total of 33, 42, 45, 17, 50, 42, 53, and 35 putative candidate CsGAox proteins were retained in LJ43, SCZ, BY, YK10, TGY, COL, CCH, and HD, respectively (**Supplementary Table 3**). Furthermore, two CsGAox proteins (ChaUn1649.3 and Cha03g006450) obtained from LJ43 in our recent study (Zhu et al., 2025), 17 CsGAox proteins identified from YK10 by Pan et al. (2017) (**Supplementary Table 3**), as well as the CsGAox proteins identified from the other seven genomes, were all aligned to the LJ43 genome. Overall, a total of 33 unique CsGAox proteins were obtained from the LJ43 genome (**Supplementary Table 1**) and used for further analysis here.

Based on the genome data of LJ43, the genetic mapping of *CsGAox* genes on the 15 chromosomes was investigated. Among 33 putative *CsGAox* genes, 27 genes were found to be unevenly located on twelve chromosomes, and six *CsGAox* genes were not assembled onto the chromosomes. The number of *CsGAox* genes on each chromosome ranged from one to five. Specifically, only one *CsGAox* gene was located on chromosomes 6, 9, 10, 11, 14, and 15, respectively. Two *CsGAox* genes were on chromosome 1. Three *CsGAox* genes were on chromosomes 3 and 7, respectively. Four *CsGAox* genes were on chromosomes 4 and 8, respectively. Five *CsGAox* genes were on chromosome 5 (**Supplementary Figure 1**).

**Figure 1:**
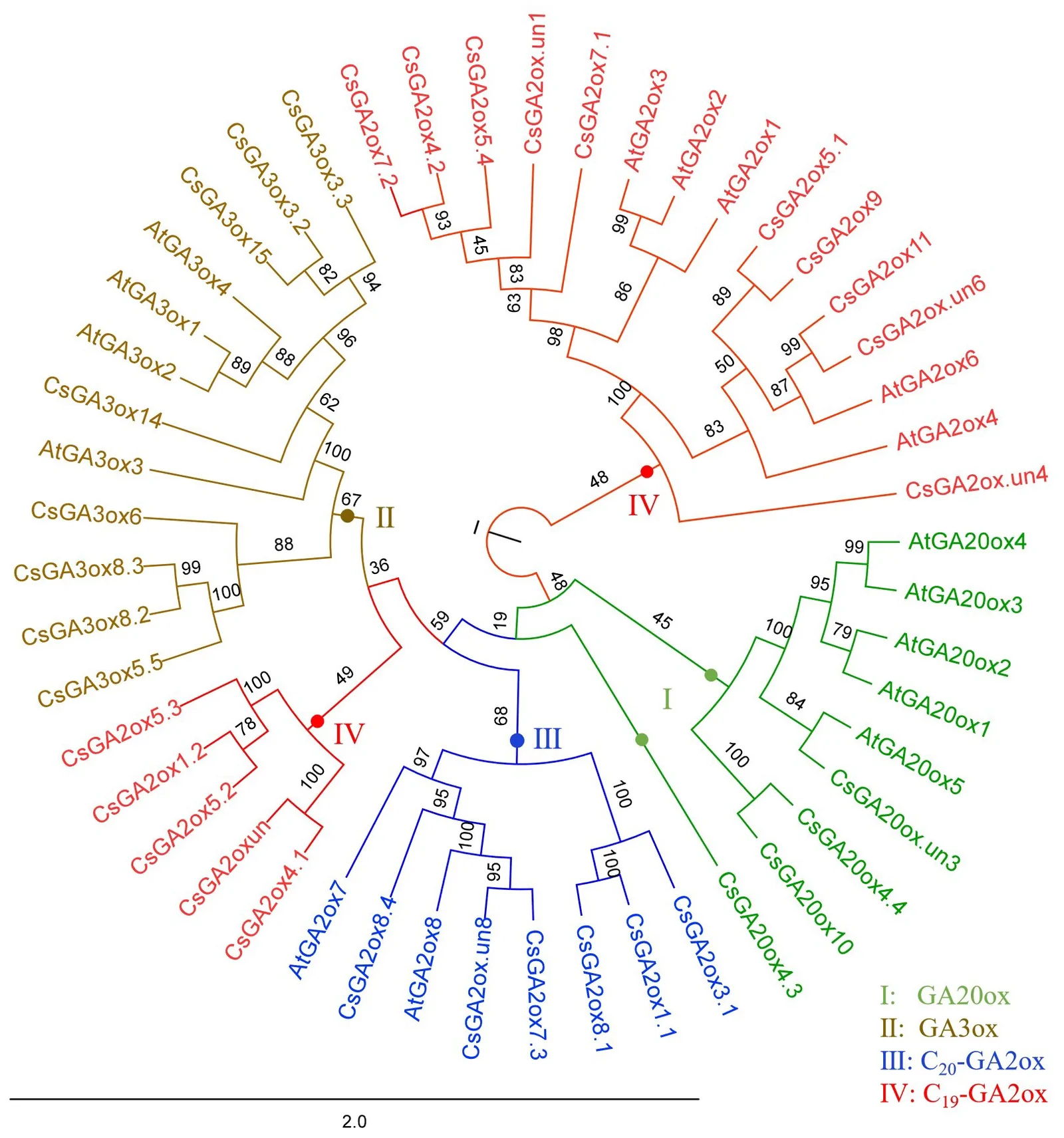
Phylogenetic relationships of CsGAox proteins from tea cv. LJ43 and AtGAox proteins from *Arabidopsis thaliana*. The maximum-likelihood (ML) tree was constructed using MEGA 7.0 under the Poisson amino acid substitution model with 1,000 bootstrap replicates and visualized using FigTree. The four subfamilies, GA20ox, GA3ox, C20-GA2ox, and C19-GA2ox, are indicated by different colors.

To further investigate the evolutionary relationships of the *CsGAox* family genes, the ML phylogenetic tree was constructed using the sequences of 33 CsGAox proteins from LJ43 together with 16 AtGAox proteins from *Arabidopsis*. According to the four AtGAox subfamilies in *Arabidopsis*, the CsGAox proteins in LJ43 were clustered into four subfamilies, namely CsGA20ox, CsGA3ox, C_19_-CsGA2ox, and C_20_-CsGA2ox. Specifically, the CsGA20ox subfamily contained four CsGA20ox proteins with five *Arabidopsis* members (I), the GA3ox subfamily contained eight CsGA3ox proteins with four AtGA3ox members (II), the C_20_-GA2ox subfamily contained six C_20_-CsGA2ox proteins with two C_20_-AtGA2ox members (III), and the C_19_-GA2ox subfamily contained 15 C_19_-CsGA2ox proteins with five *Arabidopsis* members (IV), respectively (**Figure 1**; **Supplementary Table 3**). The gene structure information of the 27 *CsGAox* genes that were located on chromosomes can be seen in **Supplementary Table 3**. These classifications were further supported by gene structure and conserved motif analyses. The *CsGAox* genes were named based on their chromosomal locations and the subfamilies to which they belong. The identified *CsGAox* genes for which loci could not be determined were named as *CsGAox.un* genes. Thus, the names of the 33 *CsGAox* genes in LJ43 were determined, which represent the location on the chromosome and their subfamilies. The numbers of *CsGAox* genes identified from the eight Camellia genomes are shown in **Supplementary Table 3**.

### 3.2 Physicochemical property analysis of CsGAox proteins in LJ43

The physicochemical properties were predicted. The detailed information and the physicochemical properties of the putative CsGAox proteins in LJ43 were listed in **Supplementary Table 1**. The CDS lengths ranged from 351 bp (*CsGA2ox7.2*) to 1326 bp (*CsGA3ox15*), encoding proteins ranging from 116 to 441 AA. The expected MW ranged from 13.40 to 49.11 kDa. The theoretical pI showed a wide range, varying from 4.76 (CsGA2ox1.1) to 9.92 (CsGA3ox3.3). Among them, 26 are acidic and seven are basic in nature. The GRAVY values of the CsGAox proteins ranged from −0.626 to −0.117, indicating that they are hydrophilic in nature. Twelve CsGAox proteins had instability indices below 40, indicating that they were relatively stable. However, the instability indices of the other 21 CsGAox proteins exceeded 40 (40.03–56.25), indicating that they were unstable.

The subcellular localization of the proteins could represent their variable functions. It was predicted that CsGA2ox7.3 might be located in the cytoplasm, nucleus and plastid, while CsGA20ox.un3, CsGA3ox3.2, as well as CsGA2ox11, CsGA2ox.un1, CsGA2ox.un6, and CsGA2ox3.1 might be located in the nucleus. Except for the above seven CsGAox proteins, the remaining CsGAox proteins were all localized in the cytoplasm and nucleus (**Supplementary Table 4**). It should be noted that 23 CsGA2ox proteins had nuclear localization signals, CsGA3ox8.2 had a peroxisomal targeting signal, while another nine CsGAox proteins had no localization signals.

### 3.3 Multiple sequence alignment and phylogenetic analysis of CsGAox family proteins in LJ43

**Figure 2:**
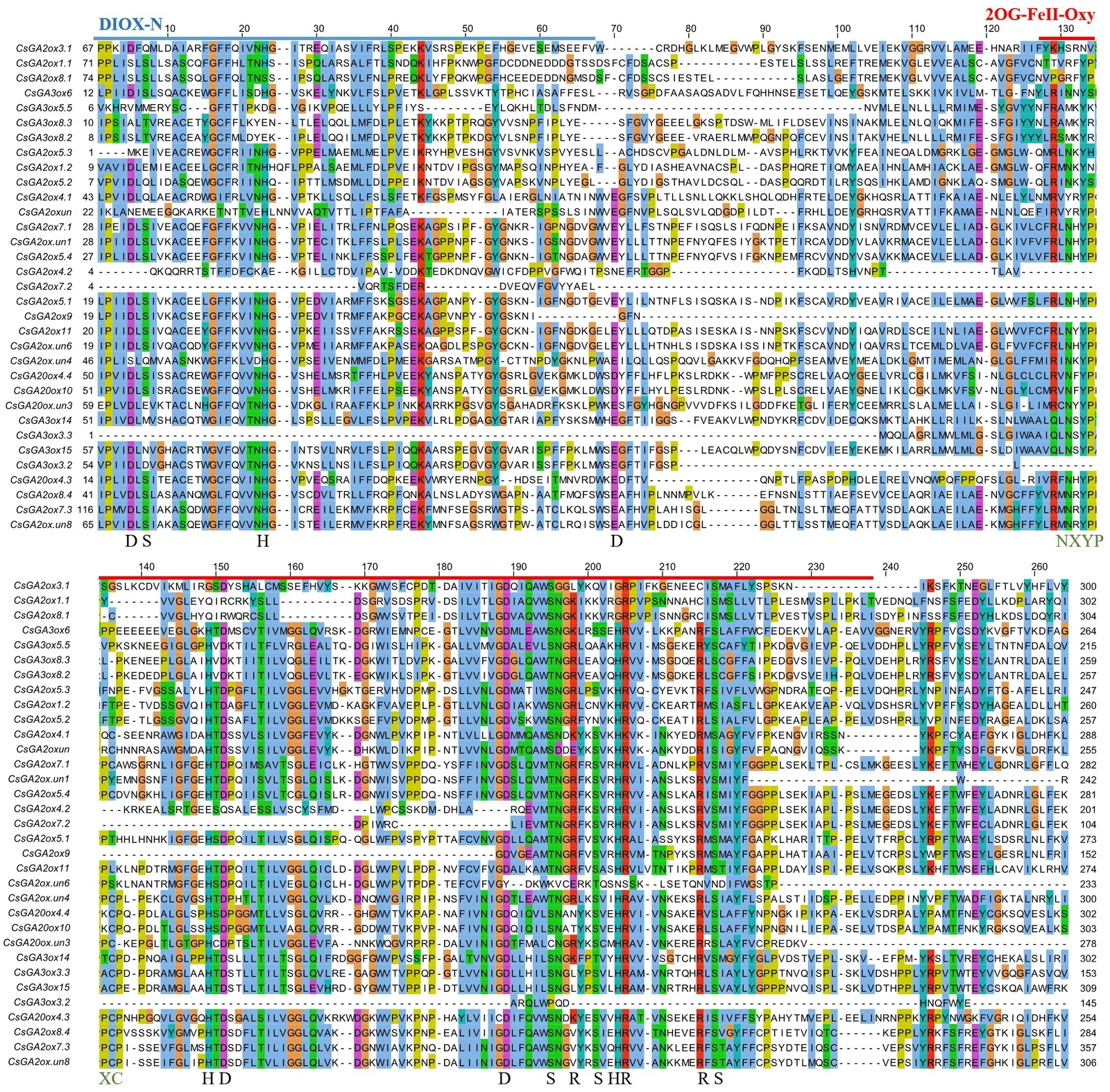
Multiple sequence alignment of 33 predicted CsGAox proteins in LJ43. The DIOX_N (PF14226) and 2OG-FeII_Oxy (PF03171) domains and the proposed consensus sequence NXYPXC are highlighted with bold lines. Three H, three R and four S residues are conserved in all CsGAox proteins, whereas four D residues are identified, with the second D residue present only in CsGA2ox7.1, CsGA2ox.un1, and CsGA2ox5.4. These conserved residues are indicated below the alignment.

Multiple alignment analysis was performed to investigate the homologous domain sequence features of 33 CsGAox proteins in LJ43. The results showed that two conserved domains, 2OG-FeII_Oxy (PF03171) and DIOX_N (PF14226), were common in all 33 CsGAox sequences. The proposed consensus sequence NXYPXC of 2-ODDs was also highly conserved in all CsGAox members. Four D residues were identified, and it was found that the second D residue only existed in the sequences of three C_19_-GA2ox proteins (CsGA2ox7.1, CsGA2ox.un1, and CsGA2ox5.4). Three H residues were conserved in all putative CsGAox proteins. On the other hand, three R residues and four S residues were identified in the sequences of CsGAox proteins (**Figure 2**).

### 3.4 Phylogenetic, conserved motifs, and gene structure analyses of *CsGAox* proteins in LJ43

**Figure 3:**
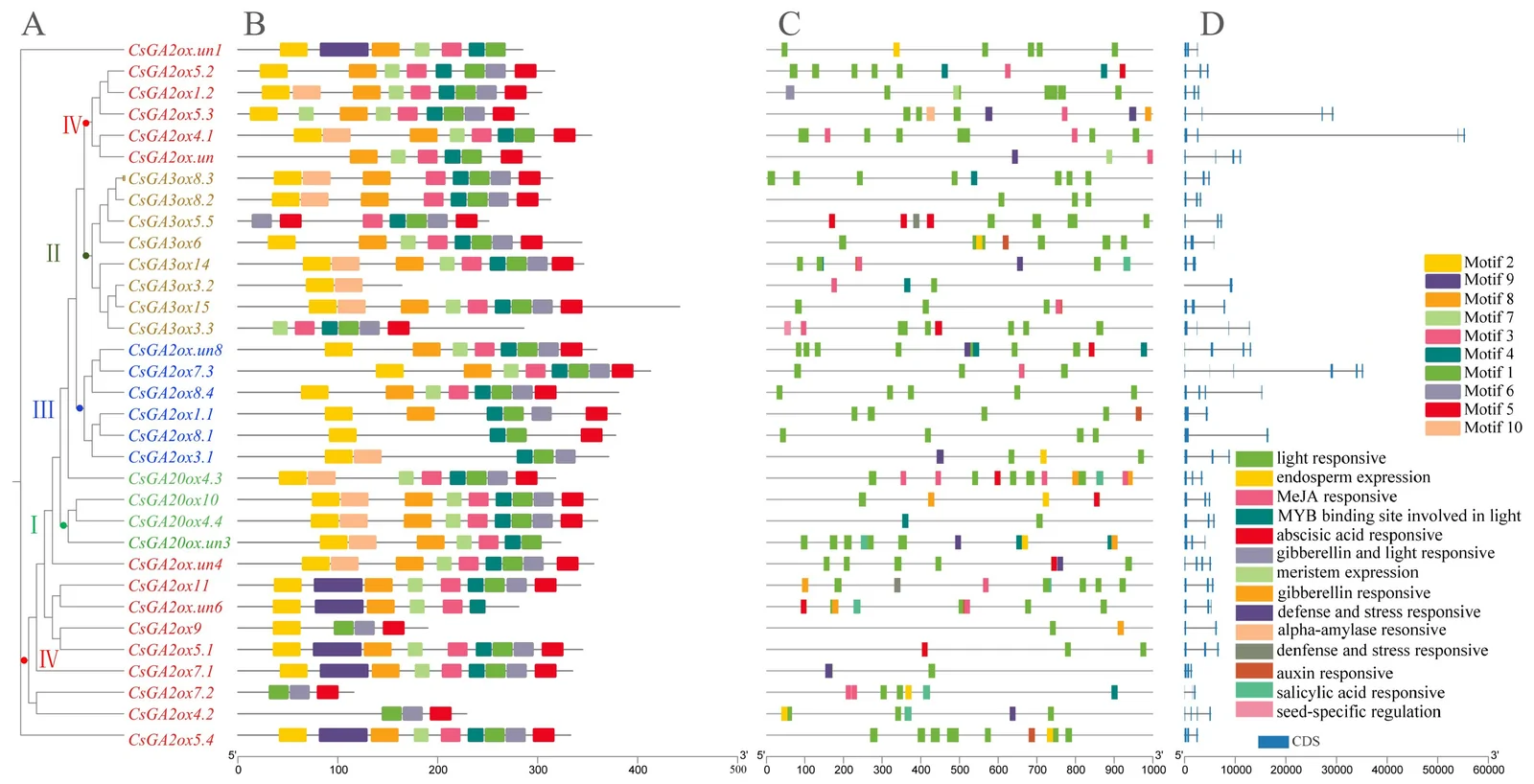
Phylogenetic relationships, conserved motifs, *cis*-acting elements, and gene structures of 33 *CsGAox* genes in LJ43. **(A)** Maximum-likelihood phylogenetic tree of CsGAox proteins. Subgroups I–IV represent GA20ox, GA3ox, C20-GA2ox, and C19-GA2ox, respectively. **(B)** Conserved motif analysis of CsGAox proteins; different motifs are indicated by different colors. **(C)** Distribution of putative cis-acting elements in the 1,000-bp promoter sequences of 33 *CsGAox* genes. **(D)** Gene structures of *CsGAox* genes; exons and introns are represented by blue boxes and dark gray lines, respectively.

A phylogenetic tree was constructed, and ten conserved motifs were identified among the 33 putative CsGAox proteins of LJ43. First, the CsGA20ox subfamily (subgroup I) contained four members, of which CsGA20ox10 and CsGA20ox4.4 each contained nine motifs (motifs 2, 8, 10, 7, 3, 4, 1, 6, and 5). Compared with the above two CsGA20ox proteins, CsGA20ox4.3 and CsGA20ox.un3 lack one (motif 8) and two motifs (motifs 6 and 5), respectively. Firstly, it can be seen that CsGA20ox proteins in subgroup I all contain motifs 2, 10, 7, 3, 4, and 1. Secondly, CsGA3ox proteins in subgroup II have eight members. To be specific, CsGA3ox14 and CsGA3ox15 have nine motifs (motifs 2, 8, 10, 7, 3, 4, 1, 6, and 5); CsGA3ox6 has eight motifs, which lacks motif 10 compared with CsGA3ox14 and CsGA3ox15; CsGA3ox8.2 and CsGA3ox8.3 contain eight motifs, both lacking motif 7 compared with CsGA3ox14 and CsGA3ox15; CsGA3ox5.5 and CsGA3ox3.3 contain seven and six motifs, respectively, and the identity and order of the motifs in these proteins are also quite different from those in the other CsGA3ox proteins. CsGA3ox3.2 only contains two motifs, which may be due to the incomplete genome assembly in LJ43 (**Figures 3A, B**).

Thirdly, C_20_-CsGA2ox proteins in subgroup III contain six members, among which three CsGA2ox proteins (CsGA2ox.un8, CsGA2ox7.3, and CsGA2ox8.4) all have eight motifs (motifs 2, 8, 7, 3, 4, 1, 6, and 5). Compared with these three proteins, CsGA2ox1.1, CsGA2ox8.1, and CsGA2ox3.1 have six motifs (lacking motifs 7 and 3), four motifs (lacking motifs 8, 7, 3, and 6), and five motifs (lacking motifs 7, 3, 8, and 5, but containing motif 10), respectively. In conclusion, C_20_-CsGA2ox proteins in subgroup III all contain motifs 1, 2, and 4. Fourthly, C_19_-CsGA2ox proteins in subgroup IV have 15 members, among which four members (CsGA2ox11, CsGA2ox5.1, CsGA2ox7.1, and CsGA2ox5.4) all have nine motifs (motifs 2, 9, 8, 7, 3, 4, 1, 6, and 5); CsGA2ox.un4 and CsGA2ox1.2 also contain nine motifs, with motif 10 present but motif 9 absent compared with the above four proteins; Compared with CsGA2ox.un4 and CsGA2ox1.2, CsGA2ox4.1 and CsGA2ox5.2 both contain eight motifs, which lack motif 6 and 10, respectively; CsGA2ox5.3 has one more motif 7 compared with CsGA2ox5.2; CsGA2ox.un1 and CsGA2ox.un6 have seven and six motifs, respectively, lacking the last two motifs (motifs 6 and 5) and three motifs (motifs 1, 6, and 5), respectively; CsGA2ox.un lacks motifs 2 and 10 compared with CsGA2ox4.1; CsGA2ox7.2 and CsGA2ox4.2 both have three motifs (motifs 1, 6, and 5), and CsGA2ox9 has one more motif (motif 2) compared with CsGA2ox7.2 and CsGA2ox4.2 (**Figures 3A, B**). These motifs are typical for GAox family proteins.

It was shown that the coding sequences of 33 *CsGAox* genes in LJ43 are all disrupted by introns, and the number of exons varies from two to six. Overall, the number of exons in most *CsGAox* genes is between two and three. Furthermore, it can be seen that nine *CsGAox* genes, including five C_19_-*GA2ox*, three C_20_-*GA2ox*, and one *GA3ox*, contain more than three exons. Some of the C_20_-*GA2ox* and C_19_-*GA2ox* members have relatively longer introns (**Figures 3A, D**).

### 3.5 *Cis*-acting element analysis of *CsGAox* genes in LJ43

To reveal the biological processes in which *CsGAox* genes might be involved, the *cis*-acting elements were analyzed. After eliminating some elements with unknown functions, 14 kinds of important elements were selected for further analysis. The distribution of the promoter elements in LJ43 *CsGAox* genes was shown in **Figures 3A,C**. Firstly, the light-responsive elements were ubiquitous and most abundant. Secondly, some hormone-responsive elements were also widely distributed, such as the response elements of GA, methyl jasmonate (MeJA), auxin, abscisic acid (ABA), and salicylic acid (SA). Notably, the *CsGAox* family genes had 38 MeJA-responsive elements, which was greater than the number of any of the other four types of hormone-responsive elements. Thirdly, ten defense- and stress-responsive elements were identified. Fourthly, some development-related elements were also identified, including endosperm expression-related elements, meristem expression-related elements, α-amylase responsive elements, and seed-specific regulatory elements. Fifthly, MYB-binding sites involved in light responsiveness were relatively abundant (**Figures 3A,C**; **Supplementary Table 5**).

### 3.6 Gene duplication of *CsGAox* genes in tea plants

To explore the genetic diversity and formation mode of the *CsGAox* gene family, the gene duplication events of Cs*GAox* family members in eight Camellia genomes were investigated. The results showed that all 17 *CsGAox* genes in YK10 were classified as dispersed duplications. Except for YK10, the *CsGAox* genes in the other seven Camellia genomes showed four duplication types, including WGD/SD, dispersed, tandem, and proximal duplication. *CsGAox* family members are involved in 13, 15, 15, 20, 15, 16, and 14 SD/WGD events in LJ43, SCZ, BY, TGY, COL, CCH, and HD, respectively. Specifically, among the 33 identified Cs*GAox* genes in LJ43, 15 genes belong to dispersed, 13 genes belong to WGD/SD, four genes belong to proximal, and only one gene belongs to tandem duplication (**Supplementary Table 6**).

**Figure 4:**
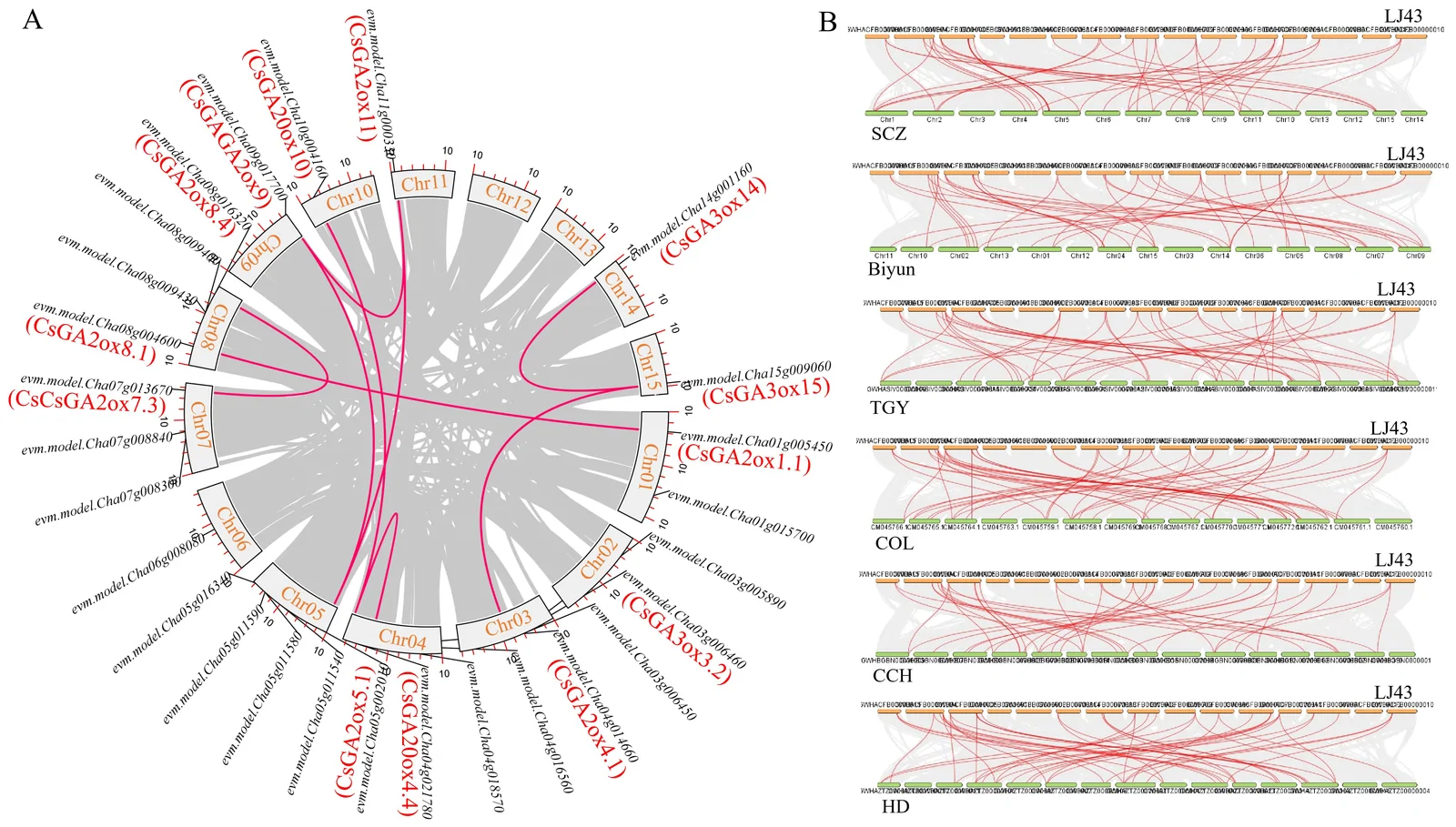
Gene duplication and synteny analyses of *CsGAox* genes. **(A)** Synteny analysis of *CsGAox* genes within the LJ43 genome. Gray lines in the background indicate collinear blocks, whereas red lines connect duplicated *CsGAox* gene pairs. **(B)** Synteny analysis of *CsGAox* genes between LJ43 and six other Camellia genomes (SCZ, BY, TGY, COL, CCH, and HD). Gray lines in the background indicate collinear blocks, whereas red lines connect syntenic *CsGAox* gene pairs. Abbreviations: CsGAox, *Camellia sinensis* gibberellin oxidase; LJ43, Longjing 43; SCZ, Shuchazao; BY, Biyun; TGY, Tieguanyin; COL, *Camellia oleifera*; CCH, *Camellia chekiangoleosa*; HD, Huangdan; Chr, chromosome.

Among the 27 *CsGAox* genes located on the 12 chromosomes of LJ43, nine duplicated gene pairs were identified, and the genes in these pairs were all associated with WGD/SD events. *CsGA2ox4.1* and *CsGA20ox4.4*, which form a gene pair, are both located on chromosome 4. However, except for this gene pair, the two genes within each of the other gene pairs are located on different chromosomes. Notably, three duplicated gene pairs are formed between any two genes of *CsGA2ox11*, *CsGA2ox5.1* and *CsGA2ox9*; *CsGA3ox15* formed two gene pairs with *CsGA3ox3.2* and *CsGA3ox14*, respectively (**Figure 4A**; **Supplementary Table 7**). To gain insights into the evolutionary constraints acting on the *CsGAox* gene family in LJ43, the *Ka/Ks* ratios of the syntenic gene pairs were calculated. The results showed that, except for BY and YK10, for which *Ka/Ks* ratios could not be obtained, all successfully calculated *Ka/Ks* ratios in the other six genomes were less than one (**Supplementary Table 8**).

### 3.7 Collinearity analysis of CsGAox genes between LJ43 and other Camellia genomes

To further explore genome synteny, comparative syntenic maps between the LJ43 genome and the other six Camellia genomes were constructed. The results suggested that a total of 22, 23, 21, 23, 22, and 23 *CsGAox* genes in LJ43 had syntenic genes in the genomes of SCZ, BY, TGY, COL, CCH, and HD, respectively. A total of 42, 41, 45, 46, 38, and 46 orthologous gene pairs were detected between LJ43 and SCZ/BY/TGY/COL/CCH/HD, respectively (**Figure 4B**; **Supplementary Table 9**). Notably, *CsGA20ox4.4* and *CsGA2ox5.1* in LJ43 were both found to have three or four syntenic gene pairs with the other six Camellia genomes, respectively; *CsGA2ox9, CsGA2ox8.1, CsGA2ox1.1, CsGA2ox11, CsGA2ox4.1, CsGA2ox7.3*, *CsGA20ox10*, and *CsGA3ox15* in LJ43 all have two, three, or four gene pairs with the other six Camellia genomes, respectively; *CsGA2ox8.4, CsGA2ox5.2*, *CsGA2ox5.4*, *CsGA3ox3.2*, *CsGA3ox14*, *CsGA3ox5.5*, and *CsGA3ox6* genes in LJ43 all have one, two, or three gene pairs with the other six Camellia genomes, respectively; *CsGA20ox4.3*, *CsGA2ox3.1*, *CsGA2ox7.1*, and *CsGA3ox8.2* genes in LJ43 all have one gene pair with the other six Camellia genomes, respectively (see the summary in **Supplementary Table 9**).

### 3.8 Association of CsGAox gene expression with branch angle formation in tea plants

**Figure 5:**
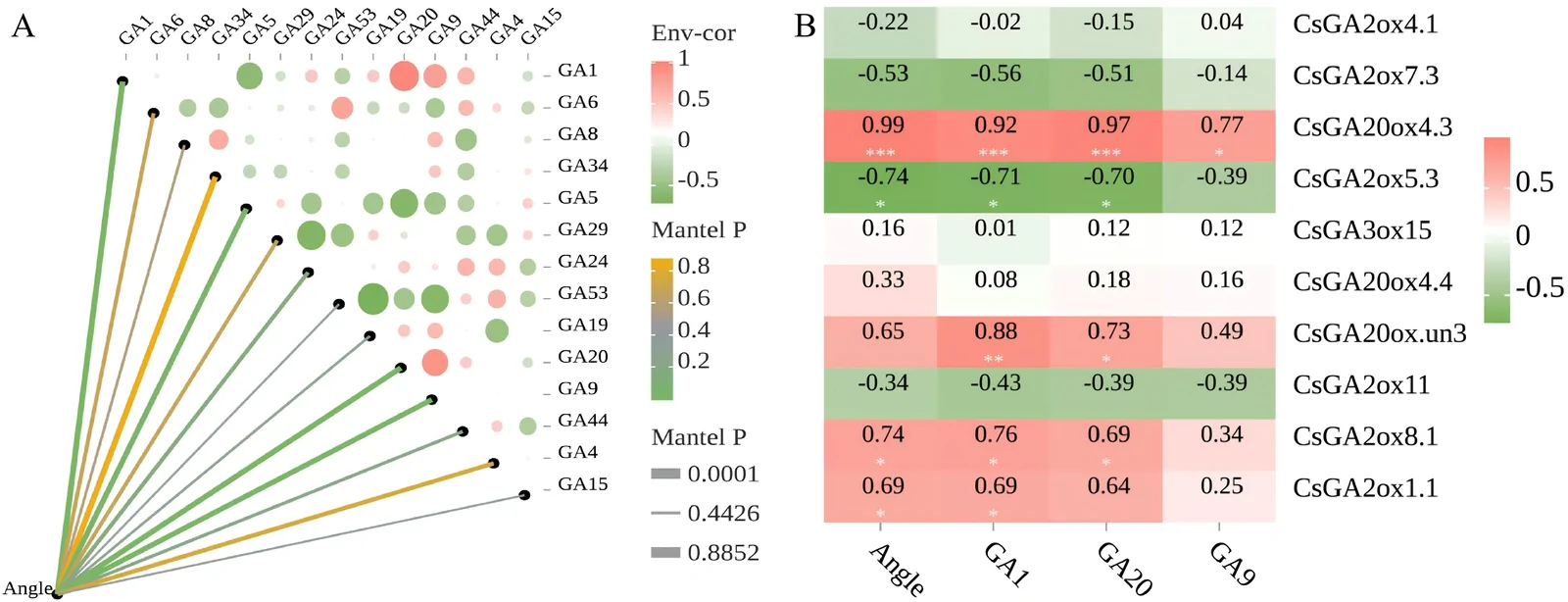
Correlation analysis among branch angle, gibberellin (GA) contents, and *CsGAox* gene expression in three tea cultivars with different branch angles: UT1 (CT2009-0025, upright tea 1), UT2 (CT2013-0114, upright tea 2), and DT (CT2009-0160, draped tea). **(A)** Correlation network heat map between branch angle and GA contents. **(B)** Correlation heat map showing the associations of branch angle and GA contents with *CsGAox* gene expression. The heat maps were generated using OmicShare Tools. Abbreviations: *CsGAox*, *Camellia sinensis* gibberellin oxidase; Env-cor, correlation coefficient among variables; Mantel *R*, correlation coefficient of the Mantel test; Mantel *P*, *P* value of the Mantel test. *, **, and *** indicate significant correlations at *P* < 0.05, *P* < 0.01, and *P* < 0.001, respectively.

In our recent report, three tea cultivars with different branch angles, including UT1, UT2, and DT, were selected to investigate branch angle formation through transcriptome and plant hormone analysis. Among the detected 18 kinds of GAs, GA_1_, GA_3_, GA_4_, and GA_7_ are bioactive GAs; the contents of GA_51_ and GA_3_ were not detected, and the contents of GA_12_-ald and GA_7_ were very low. It was found that there were higher levels of GA_1_, GA_24_, GA_19_, GA_20_, and GA_9_ in the lateral buds of the draped cv. DT when compared with two upright cvs. UT1 and UT2 (Zhu et al., 2025). Here, the results of correlation analysis showed that branch angle was highly positively correlated with the contents of GA_1_, GA_9_, and GA_20_ (**Figure 5A**; **Supplementary Table 10**). Based on the above results, three GAs, including GA_1_, GA_9_, and GA_20_, were selected for further screening of candidate *CsGAox* genes by analyzing the correlations of the branch angle or GA contents with the FPKM values of *CsGAox* genes. The results of correlation analysis suggested that, firstly, branch angle, GA_9_ content, and GA_20_ content were all highly positively correlated with the FPKM of *CsGA20ox4.3*, and the branch angle and GA_20_ content were both negatively correlated with the FPKM of *CsGA2ox5.3*. Secondly, GA_1_ content was positively correlated with the FPKM of *CsGA20ox4.3* and was negatively correlated with the FPKM value of *CsGA2ox5.3*. Thirdly, the FPKM of *CsGA20ox.un3* was highly positively correlated with the contents of GA_1_ and GA_20_ (**Figure 5B**).

**Figure 6:**
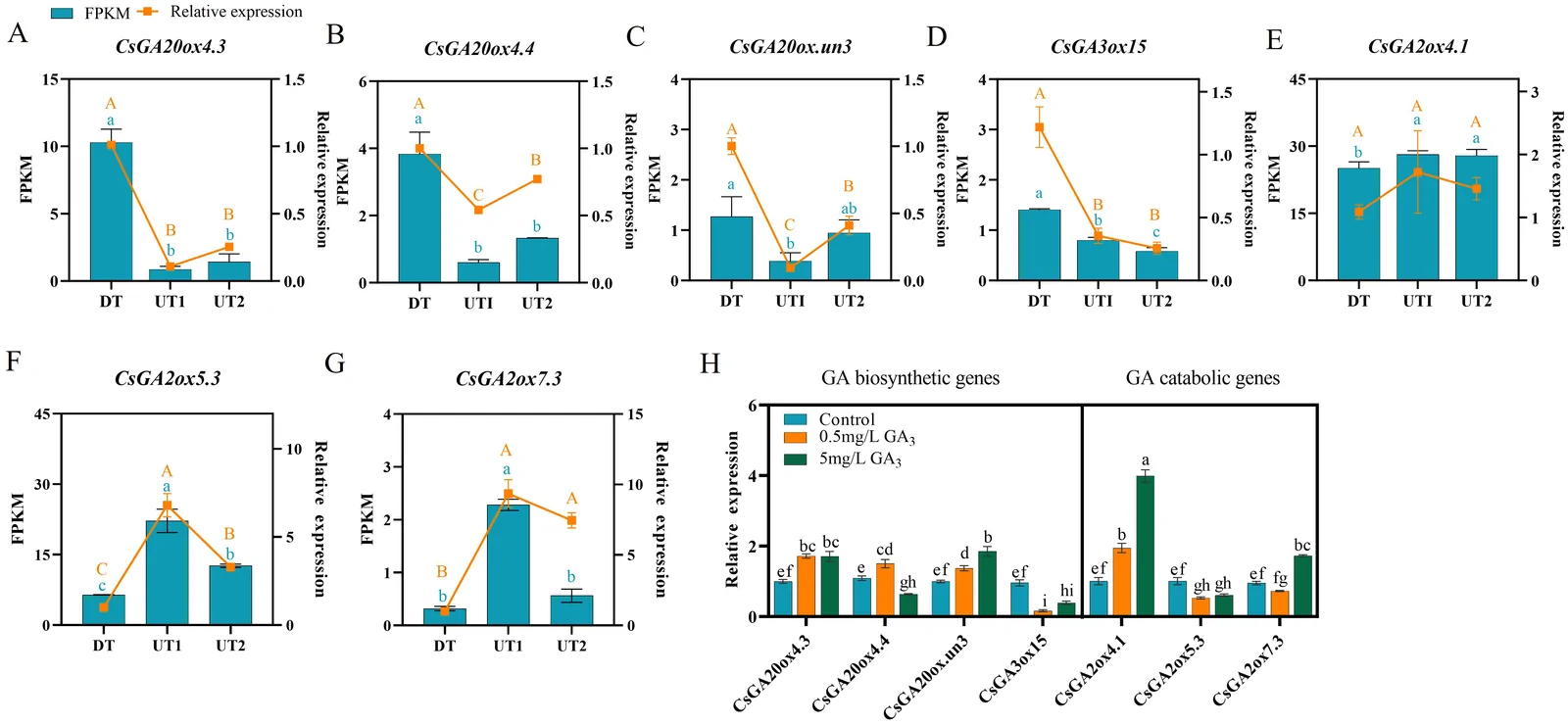
Expression patterns of *CsGAox* genes associated with branch angle in tea plants. **(A–G)** Fragments per kilobase of transcript per million mapped reads (FPKM) values and relative expression levels of seven *CsGAox* genes in the lateral buds of three tea cultivars with different branch angles: DT (CT2009-0160, draped tea), UT1 (CT2009-0025, upright tea 1), and UT2 (CT2013-0114, upright tea 2). The draped cultivar DT was used as the control. FPKM values are shown on the left y-axis, and relative expression levels determined by quantitative real-time PCR (qRT-PCR) are shown on the right y-axis. Different lowercase and uppercase letters indicate significant differences in FPKM values and relative expression levels, respectively, among the three cultivars (*P* < 0.05). **(H)** Relative expression levels of the seven *CsGAox* genes in the lateral buds of Zhongcha 108 (ZC108) under 0, 0.5, and 5 mg/L gibberellin A3 (GA_3_) treatments. Different lowercase letters indicate significant differences among all gene–treatment combinations (*P* < 0.05). Data are presented as means ± standard error (SE) of three independent biological replicates.

The FPKM values of *CsGA20ox4.3*, *CsGA20ox4.4*, and *CsGA3ox15* in the draped cv. DT were significantly higher than those in both upright cvs. UT1 and UT2, whereas the FPKM value of *CsGA20ox.un3* was significantly higher in DT than in UT1 but not UT2. Conversely, the FPKM values of *CsGA2ox4.1* and *CsGA2ox5.3* were significantly lower in DT than in both upright cultivars, whereas *CsGA2ox7.3* was significantly lower in DT than in UT1 but not UT2. Furthermore, the expression patterns of these seven CsGAox genes were further validated by qRT-PCR (**Figure 6**). The results showed that the relative expression of these seven *CsGAox* genes all showed trends similar to their FPKM values. Thus, *CsGA20ox4.3*, *CsGA20ox4.4*, *CsGA20ox.un3*, *CsGA3ox15*, *CsGA2ox4.1, CsGA2ox5.3*, and *CsGA2ox7.3* were identified as candidate *CsGAox* genes associated with branch angle variation in tea plants.

### 3.9 Exogenous GA3 treatment increased branch angle and altered *CsGAox* gene expression in tea lateral buds

Our recent report suggested that exogenous spraying of 0.5 and 5 mg/L GA_3_ both significantly increased the branch angle of tea plants. To further examine the responses of the seven candidate *CsGAox* genes to exogenous GA_3_ treatment, their relative expression levels in lateral buds were determined by qRT-PCR. Firstly, 0.5 and 5 mg/L GA_3_ up-regulated the expression of two GA biosynthetic genes, *CsGA20ox4.3* and *CsGA20ox.un3*. Secondly, 0.5 mg/L GA_3_ treatment upregulated the expression of *CsGA20ox4.4*, whereas 5 mg/L GA_3_ downregulated its expression, indicating a concentration-dependent transcriptional response to exogenous GA_3_. Thirdly, 0.5 and 5 mg/L GA_3_ both down-regulated the expression of *CsGA3ox15*. Fourthly, 0.5 and 5 mg/L GA_3_ both up-regulated the expression of *CsGA2ox4.1.* Thus, CsGA2ox4.1 showed a positive transcriptional response to exogenous GA_3_ treatment. Fifthly, both 0.5 and 5 mg/L GA_3_ treatments downregulated the expression of the GA-catabolic gene *CsGA2ox5.3*. This expression response, together with the increased branch angle following GA_3_ treatment, further supports an association of *CsGA2ox5.3* with GA-responsive branch angle formation. Sixthly, 0.5 mg/L GA_3_ treatment decreased *CsGA2ox7.3* expression, whereas 5 mg/L GA_3_ treatment increased its expression, indicating a concentration-dependent transcriptional response to exogenous GA_3_. In summary, *CsGA20ox4.3*, *CsGA2ox5.3* and *CsGA20ox.un3* were identified as candidate *CsGAox* genes associated with GA-responsive branch angle formation in tea plants.

### 3.10 Association of *CsGAox* gene expression with internode elongation in tea plants cvs. LJ43 and ZC102

During the growth of tea new shoots from the 2- to 5-leaf stage, the FPKM values of *CsGA20ox4.3* and *CsGA3ox3.3* in the internode of new shoots were both significantly positively correlated with the internode length. The FPKM value of *CsGA2ox1.1* was significantly negatively correlated with internode length (**Supplementary Table 11**). Furthermore, the FPKM value of *CsGA2ox.un1* was significantly negatively correlated with GA_3_ contents at the 2-leaf stage; the FPKM value of *CsGA20ox4.3* was significantly positively correlated with internode length as well as the contents of GA_3_ and GA_7_ at the 5-leaf stage. Subsequently, the expression patterns of *CsGAox* genes were further validated by qRT-PCR.

**Figure 7:**
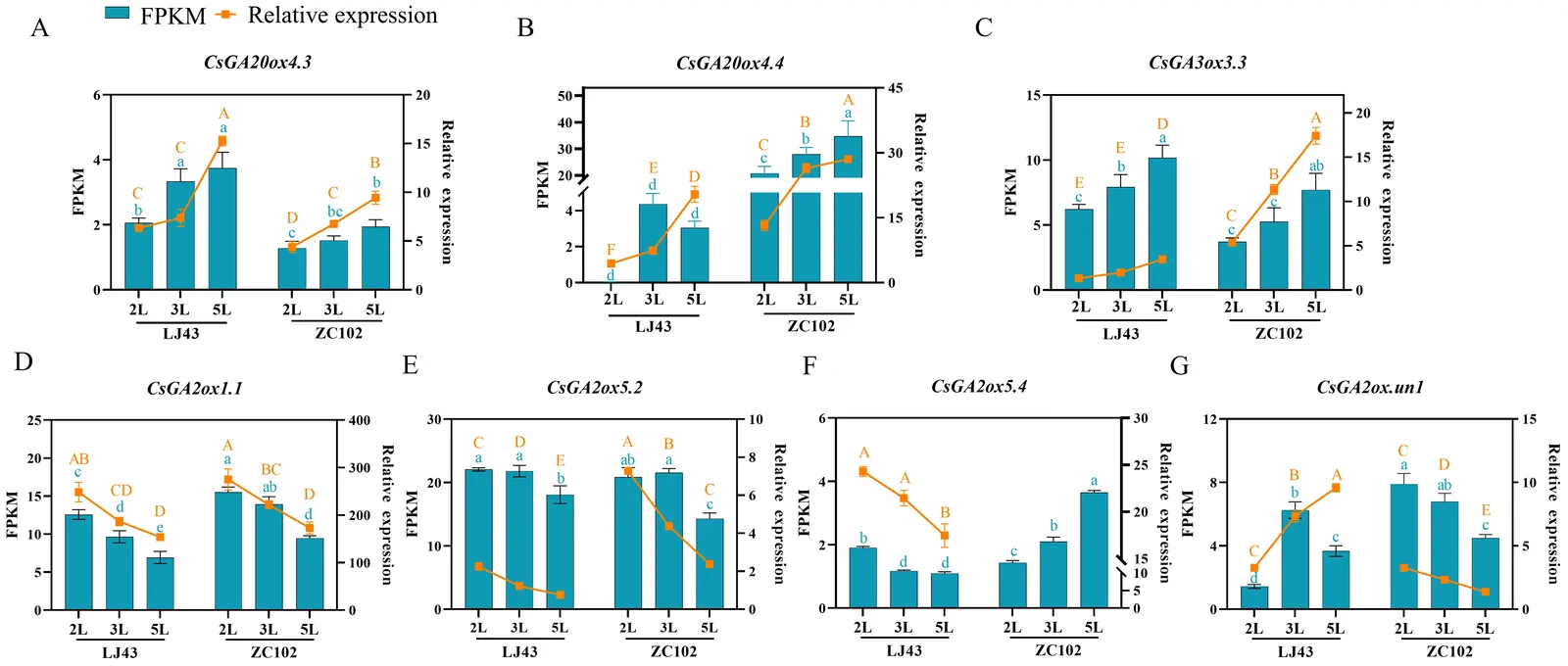
Expression patterns of *CsGAox* genes in the internodes of two tea cultivars with different internode lengths, Longjing 43 (LJ43) and Zhongcha 102 (ZC102), at the 2-, 3-, and 5-leaf stages. **(A–G)** Fragments per kilobase of transcript per million mapped reads (FPKM) values and relative expression levels of seven *CsGAox* genes. FPKM values are shown on the left y-axis, and relative expression levels determined by quantitative real-time PCR (qRT-PCR) are shown on the right y-axis. Different lowercase and uppercase letters indicate significant differences in FPKM values and relative expression levels, respectively, among the six cultivar–developmental stage combinations (*P* < 0.05). Data are presented as means ± standard error (SE) of three independent biological replicates. 2L, 3L, and 5L indicate the 2-, 3-, and 5-leaf stages, respectively.

Transcriptome results showed that with internode development, the FPKM value of *CsGA20ox4.3* showed a trend of gradual increase in two cultivars. Furthermore, the FPKM value of *CsGA20ox4.3* in LJ43 was significantly higher than that in ZC102 at the 2-, 3-, and 5-leaf stages. Similarly, with internode development, the FPKM value of *CsGA3ox3.3* showed a trend of gradual increase in the two cultivars. With internode development, the FPKM of *CsGA2ox1.1* showed a decreasing trend in the two cultivars; the FPKM values of *CsGA20ox4.3* and *CsGA3ox3.3* both gradually increased with internode development in the two cultivars; the FPKM of *CsGA2ox.un1* showed a gradually decreasing trend in ZC102. On the other hand, the FPKM values in the two cultivars at the same time point were compared. The results showed that the FPKM of *CsGA20ox4.3* in LJ43 was higher than that in ZC102 at all three time points, whereas the FPKM of *CsGA2ox1.1* in LJ43 was significantly lower than that in ZC102 at each stage. The results of qRT-PCR showed trends similar to the FPKM values of *CsGAox* genes (**Figure 7**). In addition, the relative expression of *CsGA20ox4.4* increased with internode development in both cultivars; its FPKM value increased progressively in ZC102, whereas in LJ43 it increased from the 2- to 3-leaf stage and then decreased at the 5-leaf stage. The FPKM value and relative expression of *CsGA2ox5.2* showed a trend of gradual decrease in the two cultivars, while the FPKM value and relative expression of *CsGA2ox5.4* showed a trend of gradual decrease in LJ43. In conclusion, *CsGA20ox4.3*, *CsGA3ox3.3*, *CsGA2ox1.1*, and *CsGA2ox.un1* were identified as candidate genes associated with internode elongation in LJ43 and ZC102.

### 3.11 Association of *CsGAox* gene expression with internode elongation in tea plants cvs. LJ43, QC1 and FY

**Figure 8:**
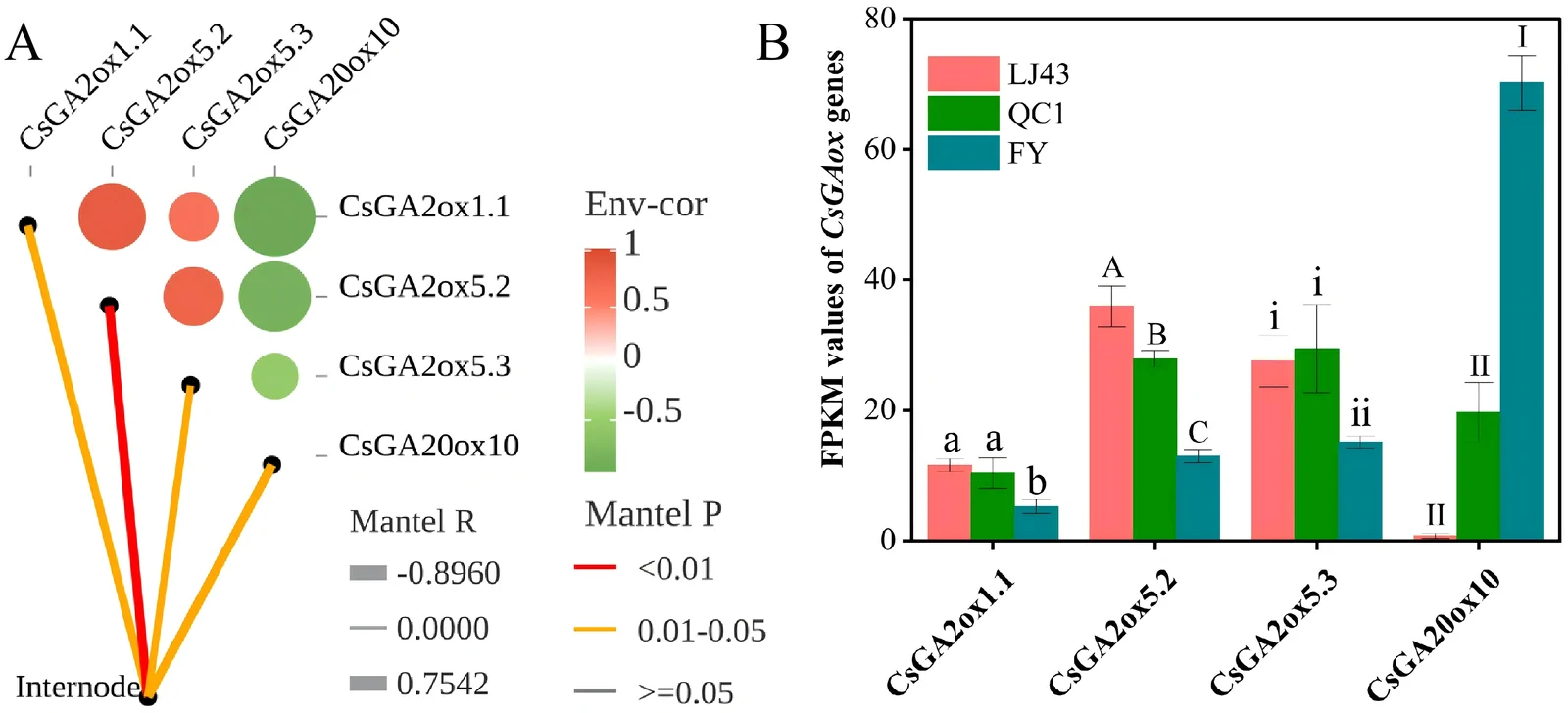
Association between internode length and *CsGAox* gene expression in three tea cultivars, Longjing 43 (LJ43), Qiancha 1 (QC1), and Feiyun (FY). **(A)** Correlation analysis between internode length and the fragments per kilobase of transcript per million mapped reads (FPKM) values of *CsGAox* genes. Circle color and size indicate the direction and strength of correlations among *CsGAox* genes, whereas line color and width indicate Mantel P and Mantel R, respectively. Env-cor denotes the correlation coefficient among variables. **(B)** FPKM values of four *CsGAox* genes in the internodes of LJ43, QC1, and FY. Different letters indicate significant differences among the three cultivars for each gene (*P* < 0.05, Duncan's multiple range test). Data are presented as means ± standard error (SE) of three independent biological replicates.

A previous report suggested that the internode length of tea cv. LJ43, QC1, and FY increased successively (Mi et al., 2025). The transcriptome data were downloaded and analyzed. The results of correlation analysis suggested that the FPKM values of *CsGA2ox* genes, including *CsGA2ox1.1*, *CsGA2ox*5.2, and *CsGA2ox5.3*, were significantly negatively correlated with internode length, while the FPKM value of *CsGA20ox10* was significantly positively correlated with internode length (**Figure 8A**; **Supplementary Table 11**). The FPKM values of these four *CsGAox* genes were displayed in **Figure 8B**. It can be seen that, firstly, there were no significant differences in the FPKM values of *CsGA2ox1.1* and *CsGA2ox5.3* between LJ43 and QC1, and their FPKM values in LJ43 and QC1 were significantly higher than those in the internode of FY. Secondly, the FPKM values of *CsGA2ox5.2* decreased successively in the internode of LJ43, QC1, and FY. Thirdly, different from the above three GA degrading genes, the FPKM values of the GA biosynthetic gene *CsGA20ox10* increased successively in the internode of LJ43, QC1, and FY. Thus, *CsGA2ox1.1*, *CsGA2ox5.2*, *CsGA2ox5.3*, and *CsGA20ox10* were identified as candidate genes associated with internode length variation among the three tea cultivars. Based on the two internode transcriptome datasets, *CsGA20ox4.3*, *CsGA3ox3.3*, *CsGA2ox1.1*, *CsGA2ox5.2*, and *CsGA2ox*.*un1* were prioritized as candidate genes potentially associated with internode elongation in tea plants.

## 4 Discussion

GAs play crucial roles in regulating the growth and development of higher plants. *GAox* genes are crucial GA metabolic genes and play key roles in regulating GA balance in plants (Davière et al., 2014; He et al., 2025; Nagai et al., 2020; Zhu et al., 2025). A total of 33 unique CsGAox proteins were obtained from the LJ43 genome in this study.

Firstly, the proposed consensus sequence NXYPXCXXP of 2-ODDs may be involved in the binding of a common co-substrate, 2-oxoglutaric acid. It was highly conserved in all of the CsGA20ox and CsGA3ox proteins, and only a partial sequence NXYPXC was highly conserved in CsGA2ox of tea cv. YK10 (Pan et al., 2017). Compared with YK10, only the partial sequence NXYPXC was highly conserved in all CsGAox members of LJ43 here. Secondly, the sequence LPWKET may be related to GA substrate binding and was conserved in GA20ox proteins of tea cv. YK10, pumpkin and *Arabidopsis*. However, it was not found in the GA20ox proteins of LJ43. The MWSEGXT sequence may be conserved in plant GA3ox proteins (Pan et al., 2017); however, it was not found in the CsGA3ox proteins of LJ43. Thirdly, previous studies suggested that the D and H residues could bind to the active-site Fe^2+^, and the H, R, and S residues could interact with the 5-carboxylate of 2-oxoglutarate (Li Y. et al., 2021). In this study, consistent with CsGAox proteins in YK10 (Pan et al., 2017), three H, three R, and four S residues were conserved in all putative CsGAox proteins of LJ43, whereas four D residues were identified, with the second D residue present only in CsGA2ox7.1, CsGA2ox.un1, and CsGA2ox5.4 (**Figure 2**).

The *cis*-acting elements play vital roles in transcription regulation (Hu et al., 2021). In general, MeJA- and light-responsive elements were ubiquitous and relatively abundant in the promoters of *CsGAox* genes in LJ43, suggesting potential responsiveness of these genes to MeJA- and light-related signals. The presence of MYB-binding sites also suggests that *CsGAox* genes may be responsive to MYB-mediated transcriptional regulation under light-related conditions.

### 4.1 Collinearity and duplication of *CsGAox* genes within LJ43 and among Camellia genomes

*CsGA3ox3.2* and *CsGA3ox3.3* (located on Chr03), *CsGA2ox5.2*, *CsGA2ox5.3*, and *CsGA2ox5.4* (Chr05), *CsGA3ox8.2* and *CsGA3ox8.3* (on Chr08) were all very close to each other, respectively, and whether they might have similar biological functions needs to be confirmed. It is worth noting that *CsGA3ox3.2* and *CsGA3ox3.3* formed a pair of tandemly duplicated genes located within a distance of 666 bp (**Supplementary Table 3**). The results of collinearity analysis within LJ43 suggested that *CsGA2ox4.1* and *CsGA20ox4.4* belong to different subfamilies, although they formed a gene pair. Except for this gene pair, the two genes within each of the other gene pairs belonged to the same subfamily. The nine identified *CsGAox* gene pairs in LJ43 were all associated with WGD/SD events, indicating that WGD or SD may be a major force driving the expansion of the Cs*GAox* gene family and that functional divergence may have occurred following duplication. The analysis of *CsGAox* duplication events revealed numerous collinear regions between LJ43 and the other seven Camellia genomes. The results showed that, except for YK10, the WGD/SD, dispersed, tandem, and proximal duplications may play important roles in the expansion of the *CsGAox* gene family in seven Camellia genomes, including LJ43, SCZ, BY, TGY, COL, CCH, and HD. However, the *GAox* genes in YK10 were classified only as dispersed duplications.

The *Ka/Ks* ratios of gene pairs can be used to explore the evolutionary constraints and the trend of gene divergence after duplication (Sun et al., 2023). Here, except for BY and YK10, the *Ka/Ks* ratios of the analyzed gene pairs were all less than one in the other six genomes, suggesting that these duplicated gene pairs have predominantly evolved under purifying selection. Many *CsGAox* genes in LJ43 shared more than one orthologous gene pair with the other Camellia genomes. It can be speculated that these genes may have contributed to the expansion of the *CsGAox* family during Camellia evolution and that these orthologous relationships may have arisen before the divergence of the analyzed lineages.

### 4.2 Associations of *CsGAox* genes with tea plant architecture

In tea production, the branch angle and internode length of newly formed shoots are two key traits affecting the integrity rate of tea bud leaves during mechanical-picking (Luo et al., 2023; Zhu et al., 2025; Mi et al., 2025). The bioactive GAs play important roles in regulating internode elongation and branch angle of plants (Davière et al., 2014; He et al., 2025; Nagai et al., 2020; Zhu et al., 2025). *GA20ox*, *GA3ox*, and *GA2ox* genes encode key enzymes involved in the later stages of GA biosynthesis and catabolism. Identifying candidate *CsGAox* genes associated with branch angle and internode length may provide a basis for future functional studies and genetic improvement of tea plant architecture.

Here, the results of correlation analysis between GA contents and the *CsGAox* FPKM values suggested that, firstly, GA_9_ and GA_20_ are both the direct products of GA20ox and the substrates of GA3ox and GA2ox enzymes. The branch angle as well as the contents of GA_9_ and GA_20_ were all highly positively correlated with the FPKM value of *CsGA20ox4.3*, and the branch angle and GA_20_ content were both negatively correlated with the *CsGA2ox5.3* FPKM value. Secondly, as a direct product of the GA3ox enzyme, an indirect product of GA20ox, and also a substrate of GA2ox enzymes, GA_1_ content was positively correlated and negatively correlated with the FPKM value of *CsGA20ox4.3* and *CsGA2ox5.3*, respectively. Thirdly, the FPKM value of *CsGA20ox.un3* was highly positively correlated with the contents of GA_1_ and GA_20_. Based on these correlations, *CsGA20ox4.3*, *CsGA2ox5.3*, and *CsGA20ox.un3* were prioritized as candidate genes associated with branch angle in tea plants.

Exogenous application of 0.5 and 5 mg/L GA_3_ significantly increased branch angle and was accompanied by the upregulation of *CsGA20ox4.3* and *CsGA20ox.un3* and the downregulation of *CsGA2ox5.3*, whereas *CsGA2ox7.3* showed a concentration-dependent transcriptional response. These coordinated phenotypic and transcriptional responses further support an association of these *CsGAox* genes with GA-responsive branch angle formation. Previous studies reported that bioactive GAs could negatively regulate *GA20ox* and *GA3ox* expression and positively regulate *GA2ox* expression through feedback mechanisms (Pan et al., 2017; He et al., 2019). Consistent with previously reported feedback responses in GA metabolism, 5 mg/L GA_3_ treatment decreased the transcript abundance of *CsGA20ox4.4*, while both 0.5 and 5 mg/L GA_3_ treatments decreased *CsGA3ox15* expression in tea lateral buds. In contrast, 0.5 and 5 mg/L GA_3_ treatments increased *CsGA2ox4.1* expression in tea lateral buds. Previous studies suggested that the expression of *GA20ox* and *GA3ox* genes plays important regulatory roles in plant stem and internode elongation. For example, the maize *ZmGA3ox* mutant *dwarf1* exhibited a dwarf phenotype (Song et al., 2011), and miRNA-mediated suppression of *ZmGA20ox3* and *ZmGA20ox5* reduced GA levels and produced a short-statured maize ideotype (Paciorek et al., 2022). The rice *OsGA3ox2* mutant *d18* exhibited a severe dwarf phenotype, while the *OsGA20ox2* mutant *sd1* showed a semi-dwarf phenotype due to decreased active GA contents in the elongating stem (He et al., 2025). In rice, deepwater conditions promoted internode elongation by increasing *GA20ox2* expression and activity in the internode (Davière et al., 2014; Nagai et al., 2020). Overexpression of *ZmGA20ox1* or *PsGA3ox1* increased the length of maize stems and pea internodes, respectively (Paciorek et al., 2022; Ren et al., 2023). On the other hand, upregulation or overexpression of *GA2ox* in various plants reduces endogenous bioactive GA levels, resulting in plant dwarfing and internode shortening (Luo et al., 2023; He et al., 2025; Mi et al., 2025; Cao et al., 2024). Consistent with these findings in other plant species, the integrated FPKM and qRT-PCR analyses identified *CsGA20ox4.3*, *CsGA3ox3.3*, *CsGA2ox1.1*, *CsGA2ox5.2*, and *CsGA2ox.un1* as candidate genes associated with internode elongation in tea plants.

Several limitations of the present study should be acknowledged. The prioritization of the candidate CsGAox genes in this study was mainly based on comparative genomic analysis, expression profiling, correlation analysis, endogenous GA contents, and responses to exogenous GA3 treatment, and their direct biological functions have not yet been experimentally validated. Further studies using overexpression, gene silencing or gene editing, together with enzyme activity assays, are required to clarify their specific roles in tea plant architecture.

## 5 Conclusions

Overall, this study provides a comparative genomic framework for the CsGAox family across multiple Camellia genomes and identifies several candidate *CsGAox* genes associated with branch angle and internode elongation. These genes represent candidates for future functional studies and further evaluation in tea plant architecture breeding, particularly for improving the suitability of tea plants for mechanical harvesting.

## Data Availability

The datasets presented in this study can be found in online repositories. The names of the repository/repositories and accession number(s) can be found in the article/**Supplementary Material**.
