# Supplementary material for: Genome-wide identification and integrated analysis of gibberellin oxidase genes associated with branch angle and internode elongation in tea plants (Camellia sinensis)
Source: Front Plant Sci. 2026 Sep 3;17:1940210. doi: 10.3389/fpls.2026.1940210 (PMC13582540; doi:10.3389/fpls.2026.1940210)
Supplement: Supplementary file 1 [file DataSheet1.zip › Supplementary Files/Table S2.docx]

**Table S2. Transcript sequences of *CsGAox* genes in LJ43 and protein sequences of CsGAox and Arabidopsis AtGAox genes used in this study.**

**List of transcript sequences of putative *CsGAox* genes in tea cultivar LJ43**

>Cha01g015700, *CsGA2ox1.2*

ATGGCCACAAACAATCAATGTAATGTTGCAGTGATTGATCTGGAAGACTTTCCAGGGCAGTCAAGGAAGATGATAGAGGCATGTGAGGAATTGGGTTGTTTCAGGATCACAAACCACCATCAATTCCTCCCTCCGGCACTGTCGGCGGAGATGAAGGCGGTGGTCCGGTCACTCTTCGACCTTCCGGTCGAAATCAAGCGGCTGAACACCGACGTCATCCCCGGCAGTGGGTACATGGCACCTAGCCAAATAAATCCTCATTATGAAGCCTTTGGGCTTTATGATATAGCCTCTCATGAAGCTGTTAATGCCTTTTGTTCTCCCTTAGATGCCTCTCCTCACCAAAGAGAAACTATACAGATGTATGCTGAAGCAATACACAATCTAGCGATGCATATTGCTTGCAAGCTAGCTGAAGGTATGGGGTTAACTAGCTTTTCGTTTGAGGGATGGCCTTGTCAATTTAGGATGAACAAATATAACTTCACCCCCGAGACTGTAGACTCTTCCGGTGTGCAAATACATACTGATGCAGGGTTCCTCACTATACTTCAAGAGGATGAAAATGTTGGCGGTCTTGAAGTCATGGACAAGGCTGGCAAATTTGTTGCAGTGGAGCCTTTGCCGGGAACTCTCCTTGTCAATCTCGGAGACATTGCTAAGGTATGGAGTAATGGTAGATTATGCAATGTGAAGCATAGAGTAGTATGCAAGGAAGCAAGAACAAGAATGTCAATTGCTTCATTTCTGTTGGGACCAAAGGAGGCAAAAGTGGAAGCTCCCGTGCAATTAGTGGATTCCTCACACTCACGCTTGTATGTTCCTTTCTTTTACAGTGATTACAGAAAGCTCCGAATCTCAAAAGAGTTACATGCTGGTGAAGCTCTTGATCTTTTACACACCAACTCTTGA

>Cha01g005450, *CsGA2ox1.1*

ATGGCGTCCTCAACTCACCACTTACCAAACCCCTACGGCACCACTGCAGCTCCACCACCGACACCCTCAACCCAACCCAATCCCCTCCCTTCCTCCGCCCCCTCCGCCGACGTTTTATCTCGTCTGCTCCACCGCCTCCCTCCCACTCTCTCCCTCCCTACCCGCCTCTCTCCTCCACCAACCACCATCACCACCACCACCACCACCTCTCCTCCTCTCATCTCTCTCTCCCCCCAAAACCCTACCCTCCTCACCGACCTCCTCTCCGCCTCTTGCCAATTCGGATTCTTCCACCTCACCAACCACTCCATTTCTCCCCAACTCGCTCGCTCCGCCGAGTCAGACTCCCTCTCTCTCTTCACCCTCTCGAATGATCAAAAACAAATCCATTTCCCCAAAAACTGGCCGCTTGGCTTTGACTGCGATGACAACGAGGATGATGACGGAACGAGTTCTGACTCGTTCTGTTTCGACTCGGCTTGTTCTCCCGAGTCGACCGAGTTGTCATTATCTTCGCTTCGTGAGTTCACTCGTGAAATGGAGAAGGTAGGATTGGAGGTGGTCGAAGCGCTATCGTGCGCGGTGGGGTTTGAGAACCCGGTCCGGGAGGACCCCACTCGGGTCTGTTCGTTGATGTGGATCTCGGAAGGGTCTCCGGGTAACACTACGGTTAGGTTTTACCCGTATGTAGTCGGGTTAGAGTACCAAATCAGGTGCCGGAAGTACTCATTGCTGGCGGATTCGGGTCGGGTTTCGGACTCGCCTCGGGTCGACTCGATCCTGGTCACACTCGGCGACATTGCTCAGGTGTGGAGCAATGGAAAGATAAAGAAAGTAAGAGGAAGACCAGTTCCAAGTTTTGAAGATGGCAGCAACAACAACAATGCACACTGCATATCAATGTCGTTGCTGGTAACTCTTCCTCTAGAAAGCATGGTCTCTCCCCTCCTTCCAAAGCTCACTGTTAATAATGGTGAAGAGGACAACCAGCTCAACAAAGAAGACGATGATGATAATGGTGATACTAGTGCAGATACAAGAGAAGATGGGGCGTTCAAATTCAATTCATTTTCTTTCGAGGATTATGCGTGGAGAATCTACCATGAACGCCTCCTCCTTAAGGACCCTCTTGCTAGATACCAAATATAA

>Cha04g014660, *CsGA2ox4.1*

ATGCCTCCATCTACCACCAACACCACCACTCACTATGACTCCTACCCACCAGTGTTCCGCCCAAAGATCGCCTCAATCCACCACTCAGCACCCACTTCACGCAACCCATACTCAGACTCAGACCCACTACCAGTAATCGACCTCCAATGCCTGAACAAGAACAAACTAGCCGAGGCTTGCAGAGACTGGGGCATATTTCGATTGGTGAACCATGGTGTCCCAACAAAGCTGCTGAGCCAGCTTCATGAGCATGCCAAGGACCTTTTCTCTCTCTCCTTTGAGACCAAACAAAGGCATGGTTTTGGAAGTCCTATGTCTTACTTTTGGGGTACCCATGGTTTAACCCCTAGTGGGGTGGCCATTGAGAGGGGTCTCAATATTGCTACAAACATTAACTGGGTCGAAGGTTTCAGTGTTCCTCTGACTCTACTGTCAAACCTTCTTGCTCAAAAGAAACTGTCACACCAGCTTCAAGATCATGGTGATGATGGTGATTATCCAATGCTTGATGCTTTCAGAACTGAGTTAGATGAGTATGGAAAGCACCAATCAAGATTGGCTACAACAATTTTCAAAGCAATAGCAGAGAACCTGGAGCTGGGTCGAGAACTGTCGGACTGTTATCTATCCACATCGACTGGTAACATGCGGGTTTACCGCTACCCACAATGTTCGGAGGAGAACCGAGCATGGGGGATCGACGCCCACACTGACAGCTCAGTGCTATCGATACTGTACCATGATGAAGTCGGAGGTTTTGAAGTTTACAAGGATGGTAATTGGCTCCCAGTCAAACCTATTCCCAACACTCTCGTCCTTCTCCTCGGTGATATGATGCAGGCGATGAGCAATGATAAATACAAGAGTGTGAAACATAAGGTGAAGATAAACAAGTACGAGGATCGAATGTCGGCTGGTTACTTCGTGTTCCCTAAGGAAAACGGTGTGATTCGGAGCTCAAACTACAAGCCTTTTTGTTACGCTGAATTTCGAGCACAAGTTCAACATGACTTAAAGACCGTTGGATACAAAATTGGTCTCGACCACTTCAAGCTTCACTGA

>Cha04g018570, *CsGA20ox4.3*

ATGGAAGAGAGCAAAGCCACAACAACCAAAGACAACAACATTCCATTGATAGACCTGTCTCTGCTAAACTCTCCAAACAACTCTTCTTCAGCCGACTCCATTGCAAGCCTAGCAGCCGAGATAACCGAAGCCTGTGAGACCTGGGGTTGCTTCCAAGTCATCAACCACGGCGTGCCGGTCGAGCAGAGCCGAGCAATCGAGTCGGCGTCGAGGCAGTTCTTTGATCAGCCGAAAGAAGAGAAGAAGAAAGTGTGGAGGTACGAGAGGAATCCATTGGGGTACCATGATTCGGAGATTACGATGAATGTGAGGGACTGGAAAGAGGTGTTTGATTTCACAGTCCAAAATCCCACTCTCTTTCCAGCTTCCCCTGATCCCCATGACTTGGAACTGAGGGAGTTGGTTAATCAGTGGCCCCAATTTCCTCCTCAATTTAGCTTAGGATTGCCAAGACGAAGGTTGAATCACTTCTTCAAAGACCAAACCAGCATTGTTAGGTTCAACCACTACCCACCTTGCCCTAACCACCCTGGTCAAGTTCTTGGTGTTGGCCAGCACACTGATTCCGGAGCCTTATCCATCTTGGCTCAAGATGATGTTGGAGGATTGCAAGTGAAGCGAAAATGGGATGGAAAGTGGGTTCCTGTCAAACCCAACCCTCATGCCTATTTAGTTATCATTTGCGACATTTTTCAGGTTTGGAGCAATGACAAGTACGAGAGTGTGGTGCACAGGGCAACGGTGAACTCTGAAAAGGAAAGGATTTCGATTGTGTTCTTCTCCTACCCAGCACATTACACCATGGTTGAGCCTCTGGAGGAGCTAATAAACGATCGAAACCCTCCCAAATACAGACCATACAACTGGGGAAAGTTTTCTGCTGCCCGAAGGCGTAGTAATTTCACAAAGAACAATGTTGGAAGGATCCAAATCGATCATTTTAAGGTTATTTGA

>Cha05g002010, *CsGA2ox5.1*

ATGGTGGTGGCCACTCCAACCCCAATTAGAAGTGAAAAGATCAGAGAAGTTGAGCTTCCAATCATAGACCTCTCAGCAGAGAGATCAGAGGTGTCAAAGCTCATTGTAAAAGCTTGTGAAGAGCTTGGTTTTTTCAAGGTCATCAACCATGGTGTCCCTGAGGATGTTATTGCAAGGATGGAAGAAGAAAGCTTCCTGTTCTTCTCCAAATCAGGCTCCGAAAAACAACGGGCCGGGCCAGCTAATCCCTATGGTTACGGAAGTAAGAACATAGGCTTCAATGGTGATACTGGAGAGGTTGAATATCTTATTCTCAACACCAACTTTCTCTCCATTTCTCAAAGATCCAAAGCTATCTCCAATGACCCAATTAAATTCAGTTGTGCAGTGAGGGATTACGTGGAAGCGGTTAGAATTGTGGCATGTGAGATATTGGAGCTGATGGCAGAGGGATTGTGGGTCCCAGACGCATCAGTGTTTAGTAAGCTAATCAGGGACGTTGATGAGGACGACTCCCTTTTCAGGCTCAATCACTATCCTCCTCTCCTCCTCACGGACAGCTTTCAAGATAGTGACACGTCACCCTCCTCATTTCATCATCACCTTCATAATCATAAGATTGGATTTGGAGAACATTCTGACCCTCAGATCTTGACCATCTTAAGATCCAACGATGTCAGTGGGCTCCAAATCTCTCCACAACAAGGTCTGTGGTTCCCAGTTTCTCCTTACCCCACTACTGCCTTCTGTGTTAATGTGGGTGATTTGTTGCAGGCCATGACAAATGGGAGGTTTGTGAGTGTGAAACATAGAGCCCTAGCCAGCTCGTACAAGTCAAGAATGTCAATGGCCTATTTTGGTGCACCAAAACTCCATGCAAGGATCACCACGCCGCCAGAGTTGGTCACCCAGTTCAGACCATGTCTCTATAGACCCTTCACATGGGCCGAGTACAAGGAGACTACATATTCTCTCCGGCTCAGCGATAGTCGTCTCAACCTTTTCAAAGTGCAAGAGGGTGATGAAATTGTCTGA

>Cha05g011540, *CsGA2ox5.2*

ATGGGTACTAGTAGTCTCGTACCAGTGATTGATCTGCAAGACTTTCCATCTCAATCCCAGAAACTAATCGATGCGAGCCAAGAATGGGGTTGCTTCAGGATCATCAATCACCAGATCCCCACAACTCTGATGTCAGATATGAAGTCGGTGGTGAGGTCTCTTCTCGATCTTCCGCCGGAAATCAAACGCCGGAACACCGACGTCATCGCCGGAAGTGGGTACGTGGCGCCGAGCAAGGTTAATCCTCTATATGAAGGGTTGGGTCTCTATGACATTGGATCCACTCATGCTGTTCTTGATTTTTGCTCTCAGCTCGATGCTTCTCCTCAACAAAGGGACACAATACTGAGGTATTCTCAGTCTATACACAAACTAGCCATGGACATTGGGAACAAATTAGCTCAAAGTATGGGTTTGGCCAGTGATTTGTTTATGGGTTGGCCCTGCCAATTGAGGATTAACAAATACAGCTTCACCCCTGAAACTCTCGGATCATCCGGGGTTCAAATCCACACAGACTCTGCATTTCTTACCATATTACAGGATGATGAAAACGTGGGCGGTCTTGAAGTGATGGACAAGAAGTCCGGTGAATTTGTTCCGGTTGATCCCATGCCCGGCACCCTCCTTGTCAATCTTGGAGACTTTGCTACGTCGGATTTGATTGATACATTCACCAATGTGTCAAAGGTGTGGAGCAATGGAAGATTTTACAATGTGAAGCACAGAGTGCAGTGCAAGGAAGCCACGATACGGCTGTCAATTGCTTTGTTCGTCTTAGGACCAAAGGAGGCTCCACTGGAAGCGCCACCGGAGCTAGTGGATAGTGAAAGCCACCCTCGGCTCTATGTTCCAATCAATTTTGAAGATTACAGAAAGCTCCGGCTTTCCACTGGTTTGAGAGCCGGTGAAGCTCTTGACAAGTTATCTGCAAGCAATGTTCCATTTTGA

>Cha05g011580, *CsGA2ox5.3*

ATGAAAGAAGATTCTGTAGGACAATCAGAGAAAATAGTAGAAGCATGCAGGGAGTGGGGATGTTTCAGGATCATCAACCACGGCGTCCCACCGGAGTTGATGGCGGAGATGAAGGTGGCGACGCAGTCTCTGATGGAGCTCCCGGTGGAGATCAAACGGCGGCGTTATCATCCAGTGGAAAGCCATGGGTATGTATCAGTTAACAAAGTCAGTCCAGTTTATGAGAGCTTGTTGGCTTGTCATGACTCCTGTGTCCCTGGTGCTCTTGATAATTTTTTGGATCTCATGGCTGTCTCTCCTCACCTAAGGAAAACAGTAGTGAAGTACTTTGAAGCAATCAATGAGCAAGCATTGGACATGGGGAGGAAGTTGGGGGAAGGGATGGGGCTGAAAGGGGATTTGTTCAAGGATTGGAGTTCCCAAATGAGGTTGAACAAATACCATTTCAACCCTGAATTTGTAGGATCATCTGCTCTCTATCTACACACAGATCCTGGATTTCTTACTATTTTACAGGATGATGAAATTGTTGGTGGACTTGAAGTTGTGCACGGAAAGACTGGTGAACGTGTACATGTTGATCCCATGCCTGACTCCCTACTTGTCAACCTTGGAGATATGGCTACGATATGGAGCAATGGAAGACTTCCAAGTGTGAAGCACAGAGTCCAATGCTATGAAGTAAAGACAAGGTTCTCAATTGTTTTCCTTGTGTGGGGGCCAAATGACAGAGCAACTGAACAACCACCAGAGCTGGTGGATTCTCAACATCCTCGTCTCTATAATCCTATCAATTTTGCAGATTATAGATTGCTTCGACTCTCCAACAATTTATTCACTGGGGCTTTTGAGCTCTTGCGCATCAATTCCTGA

>Cha05g011590, *CsGA2ox5.4*

ATGGTGGTTTTATCTAAACCAGCAATTGAACAGTACTCTGTCATCAAAACCTCCAAACCCACCACATTCTCCTCTAGCATTCCCTTAATAGACCTATCAAAACCCGACACGAAGACCCTCCTCGTCAAGGCCTGCGAAGAGTTCGGATTCTTCAAGGTGATCAATCATGGCGTCCCAACAGAACTCATCAACAAATTGGAATCAGAAGCTTTAAAATTCTTCTCCTCACCCCTGTTTGAAAAAGAAAAAACAGGGCCTCCTAACCCATTTGGCTATGGAAACAAGAATATTGGAACCAACGGCGATGCCGGTTGGGTCGAATATCTCCTCCTAACAACAAACCCAGAATTCAATTATCAGAGATTTGTATCAATTTTCGGCAAAAACCCAGAAATGTTTCGTTGTGTTGTTAACGATTATGTGTCAGCTGTGAAGAAGATGGCTTGTGAGGTTCTAGAGTTATTGGCTGATGGGTTGAAGATTCAGCAAAGGAATGTGTTGAGTAAGCTTTTGATGGACGAACAGAGTGACTCTGTTTTCAGGCTTAATCACTACCCTCCATGCCGTGAAATCCAAGATGTGAATGGTAAGCATTTGATTGGATTTGGAGAGCACACTGACCCACAAATCATTTCTGTTCTAAGATCCAACAACACTTGCGGACTTCAAATCTCTTTGAGAGATGGGAATTGGATTTCTGTCCCTCCTGACCAGAATTCATTCTTCATCAATGTTGGTGATTCTTTGCAGGTGATGACTAATGGAAGGTTCAAAAGTGTGAGGCATAGGGTTGTTGCAAACAGTTTGAAAGCAAGGATTTCAATGATCTATTTTGGAGGACCCCCATTGAGTGAAAAGATAGCTCCATTGCCTTCACTTATGGAAGGAGAAGACAGCTTGTACAAAGAGTTTACATGGTTTGAGTACAAGACATCTGCATACAACTCAAGACTTGCAGATAATAGGCTTGGCCTCTTTGAGAAAATAGCAGCCTCATAA

>Cha06g008080, *CsGA3ox6*

ATGATGTCTGATGAATCTCATACATCTCTGCAACTACCCATTATAGATATCTCTCTGCCATTGCAATCACCATCTTCTCTCTCATCTCTCTCTCAAGCCTGCAAAGAATGGGGTTTCTTCCTCATATCCGACCATGGAGTTTCCAAAGAACTATACAACAAAGTTAGATCACTATCTGAATACCTTTTTAGCCTTCCTGTTGAGACAAAAACAAAGCTTGGTCCTTTGTCTAGTGTAAAAACATACACTCCTCATTGCATTGCCTCTGCTTTCTTTGAGAGCCTTAGGGTCTCTGGACCAGACTTTGCTGCCTCTGCTCAGAGCTCTGCAGATGTTCTCTTTCAGCACCATAATTCTGAATTCAGTGAAACACTGCAAGAATATGGGAGCAAAATGACAGAACTATCCAAGAAAATTGTGAAGATTGTGTTGATGACCTTGGGAGATGATTTTGAAAACAAGTTCAATGACTCTGAGTTCAACAACTGTCATGGGTATTTGAGGATAAACAACTATTCGCCTCCACAAATCTTGGAAGAAGAAGAAGAAGAAGAGGTTGAGGGTCTTGGAAAACACACTGACATGAGCTGTGTAACAATTGTGTATCAAGATGAGATGGGTGGTCTCCAAGTGAGATCAAAAGATGGCCGTTGGATCGAAATGAATCCTTGTGAGGGCACACTTGTGGTGAATGTGGGTGATATGCTTGAAGCTTGGAGCAATGGAAAGCTGAGATCATCTGAGCACAGAGTGGTGTTGAAGAAGCCAGCGAACCGGTTTTCTTTGGCTTTCTTTTGGTGTTTTGAGGATGAGAAAGTGGTGTTGGCACCTGAGGCAGTGGTTGGGGAAGGGAATGAGAGGGTTTATAGGCCATTTGTTTGCTCTGACTATTTGAAATTCAGAGAGACCAATGAGGAAGGAAAATTTGAGAAAGTTGGATTTACTGTTAAAGATTTTGCTGGGATTAATGCAATAGTTGAAAGCGCTTGCTTAATCATCCTTGCTGATGCCACTGCCGCTCCTCGAAACTGA

>Cha07g008300, *CsGA2ox7.1*

ATGGTGATTCTATCTCAATCAGTAGTAGAAGATTTCTCTCACATCATGACATGCAAACCAACTAGTGGCCTTTTCACTGGAATCCCAGAAATAGACCTCTCAGACCCTGATGCAAAGATCTTAATAGTTGAGGCTTGTCAAGAGTTTGGGTTCTTCAAGGTGGTCAACCATGGTGTCCCTATTGAGCTCATCACTAGATTAGAAGCTGAGTCTGTCAAGTTCTTCAACTTGCCTCAGTCTGAAAAGGACAAAGCTGGCCCTTCTATCCCTTTTGGTTACGGAAACAAGAGAATTGGCCCAAATGGTGATGTGGGTTGGGTTGAATACCTCCTCTTCAGCACCAACCCTGAATTCATTTCCCAGATGTCTCTCTCCATTTTCCAGGATAACCCAGAAATTTTCAAGTCTGCTGTGAATGATTACATCTCAAATGTAAGAGATATGGTGTGTGAAGTCTTGGAATTGATAGCTGATGGGTTGAAGATTGAGCCAAGGAATGTGTTGAGTAAGCTTTTGAGGGATGAGAAGAGTGACTCTTGTTTCAGGCTAAACCACTATCCACCATGCCCAGAGCTTCAAGCATGGAGTGGTCGAAATTTGATTGGGTTTGGAGAACACACAGACCCACAGATAATGTCTGCTGTAAGATCTAACAACACATCAGGACTTGAAATCTGTCTGAAACATGGGACATGGGTCTCAGTCCCGCCTGATCAGTACTCCTTTTTCATCAATGTCGGTGATTCCCTGCAGGTGATGACTAATGGGAGGTTTAGGAGTGTGAGGCACAGAGTTTTGGCTGACAACTTGAAACCAAGGGTGTCAATGATTTACTTTGGAGGCCCACCCTTGAGTGAAAAGTTAACACCTTTATGTTCACTAATGAAAGACGGAGAAGAAAGTTTGTACAAGGAGTTCACCTGGCATGAATACAAGACATCTGCTTACAAGACTAGGTTGGGTGACAATAGGTTGGGGTTCTTTCTGCAAAATGCAAGCCAGTGA

>Cha07g013670, *CsGA2ox7.3*

ATGCTCCCCACCCCATTTCACTCTCTCCTCCTTTCCTGTCATGTCATGGCTGGCCAAAAGTATGGTTCAATGGTTTCGAAACATCTGAGAAGACTACAAATGTCCTATGTGCCGACATGGACTCAGATGCCAGGAGACCAATGTGTACTAAAGCTAGAACACTGGTGCTCATTTGAGAAGGCAGTGATGGAGTCTGATAAATTTTCTGCAGATAGAAATCAGCCTTTGGACATGGGATCATCAGAGCCACCATTTCAAGACACTTACAAGGCTCTCTTCGACGACTACGTCGATGCAGAGTCAAAGTATGAGAAAAATGATGTTGTCATCGTTGCAGAATGTGAGCTACCGATGGTTGATCTTAGTCGGTTAAATCTCGGGGAAGAGGAGAGAGAAGATTGCAAGAGAGAGATAGCCAAAGCTTCACAAGATTGGGGGTTCTTCCAAGTGGTGAACCATGGAATTTGTCGCGAGATTTTGGAGAAAATGAGGAGAGAGCAGATGAAGGTGTTCAAGAGGCCGTTTTGTGAGAAGATCAACGACGAGTTTATGAACTTCTCCGAAGGTAGTTACCGGTGGGGAACACCGTCGGCGACTTGCCTGAAACAGTTGTCTTGGTCTGAAGCATTTCATGTTCCTTTGGCTCATATTTCTGGTTTGGGTGGTCTCACCAATCTCAGCTTGACCATGGAACAGTTTGCCACAACAGTTTCAGACCTAGCACAAAAGCTTGCAGAAATATTGGCTGAGAAAATGGGTCACAAATCCACATTTTTCAAAGAAAATTGTTTGCCAAATACTTGCTATCTCCGAATGAATAGATACCCACCATGTCCAATATCTTCAGAGGTCTTTGGATTGATGTCACACACTGATAGTGATTTCCTCACAATATTGCATCAAGATCAAATTGGTGGATTGCAATTAGTCAAAGATGGGAATTGGATTGCTGTTAAACCAAATCAAGATGCCCTAATTATTAACATCGGTGACTTATTTCAGGCATGGAGCAATGGAGTTTATAAGAGTGTTCAACACCGGGTTGTCGCCAACAAGCTAGAGGAAAGATTCTCCACCGCATATTTCTTCTGTCCATCCTATGATACTGAGATTCAAAGTTGTGTTGAGCCCTCTGTCTATAGAAGGTTTAGTTTTAGAGAATTCAGACAAGAAGTCCAAGAAGATGTCAAAAAATTTGGTTACAAAATAGGACTTCCTAGCTTTCTTGTTCAAAATTAA

>Cha08g016320, *CsGA2ox8.4*

ATGGAATCAGAACCACCATTTCAAGAAATCTACAAGACCCTTTTCGTAAACTTCTCGGAAGAAGGTACTAACTCAGAGAGTGTGCATGAGAAAAAACCTATGATCCTCGACGAATATGAGATCCCACTTGTTGATCTTAGTCGTTTAAGTCTCTGCAACTTGGAACAGAAAGAGGGCAAGAGAGAGCTTGCTAGTGCTGCAAACCAGTGGGGGCTTTTCCAGGTTGTGAATCATGGTGTTTCTTGCGATGTCTTGACAAGGTTGCAGCGCGAGCAAGTGAAGCTTTTCCGGCAACCATTTCAGAATAAAGCTTTGAACTCATTGGCAGACTATTACAGTTGGGGGGCTCCTAATGCTGCTACTTTTATGCAGTTCTCTTGGTCTGAAGCTTTTCATATTCCTCTCAACAACATGCCGGTTTTGAAAGAATTCAATAGTAATCTAAGCACAACAATTGCTGAATTTTCTGAAGTGGTTTGTGAGCTGGCTCAAAGAATAGCTGAAATATTAGCAGAGAATGTTGGGTGTAGATCCACTTTCTTTGCAAAAAATTGCCAACCTAGCTCATGTTATGTGAGAATGAACAGGTACCCACCATGCCCAGTGTCATCATCGAAAGTGTATGGGATGGTTCCACATACTGACAGTGACTTCCTCACAATATTGTGCCAAGACCATGTTGGAGGTTTGCAAGTGGCCAAAGATGGAAAATGGGTTAGTGTTAAACCCAATCCAGAGGCTTTAATTGTCATCATTGGTGATTTATTCCAGGCATGGAGCAATGGTGTGTACAAGAGCATAGAGCACAGGGTTGTGACCAATCATGAAGTAGAAAGGTTTTCTGTTGGATATTTTTTCTGTCCTACCATTGAGACTGTCATACAAACTTGCAAGGAGCCTCCATTGTACAGGAAATTTAGCTTCAGAGAGTACAGAGGACAAGTTCAAGAGGATGTCAAGACTGCCGGCACCAAAATAGGGCTTTCGAAGTTTCTCATCACCAATTTGTGTCTGTCACACCCTTCCAGTGGCCTGTCTCATCATGGTCTACCGCTCTTTAAAAGGAATCTAAATGATAACCAGATGTATCTAGCCGCAGAGGAAACAAATATCACACCTGAAACACGAAAGAACACTGAATAA

>Cha08g009400, *CsGA3ox8.3*

ATGGGTGCGGATGCGGCTGCAGAAATGATCCCATCAATTGCTCTGACGAATGAGGTGTTGAGTTTGGACAGAGAGAGCGAAGAATGGAAAGCGGTGATTAGAAAGGTGAGAGAGGCATGCGAAACATATGGGTGCTTTTTCCTTAAGTACGAGAATCTGACATTAGAGCTTCAACAACTACTGTTCAAGACCACCGGCGAGATGTTCGATCTCCCATTGGAGACCAAATTGCAGTACAAAAAACCTACTCCTAGACAAGGCGGTTACGTGGTCAGCAACCCATTTATCCCTCTCTACGAGAGCTTTGGAGTCTATGGTGAAGAAGAGTTGGGTAAAAGTAGACCTACTGACTCTTGGATGCTTATATTTTTGGATAGCGAGGTTATCAACTCCATAAATGCAAAGATGCTCGAACTGAACCTCCAAATTCAGAAAATGATTTTCGAGAGCTTTGGAATCTTAGAGTACTACAAGGAGAAGGCACTAGATGAGAACATTATTGGCAATTTACGTGCCATGAAGTACAGACTTCCTAAAGAGAATGAAGAGCCTCTTGGTCTCGCGATTCATGTTGATAAAACCATCATAACCATTTTATGTCAAAATTCAGTGCAAGGTCTTGAGATTCTCACTAAAGACGGAAAATGGATTACACTTGACGTTCCTAAGGGAGCCTTGGTAGTCTTTGTTGGCGATGGGCTCCAGGCATGGACCAATGGACGACTTGAAGCTGTGCAACACAGGGTGGTGATGAGTGGTGATCAAGAGAGGCTTTCGTGCGGATTCTTTGCTATTCCAGAGGATGGAGTGAGTATTGAGGTGCCTCCACAGCTTGTAGATGAAGAACATCCTCTCCGCTACCGATCATTCGTTTATTCTGAGTATCTTGCCAAATACCTTGCCAATACTAGGTTGGATGCGCTCGAGATTTATGCCGGTGTTTGA

>Cha08g004600, *CsGA2ox8.1*

ATGGCGTCCTCCGCCCACGACCAACTCCACCAGCTACCGAACTCTAATTACGGCACCACCGCAGCACCGCCGCCGACACCCTCCACACAGCCCAACCCCTACTCCTCCTCAGACCTCGCCGCCGACGTCCTCTCTCGACTTCTCCACCGCCTCCCTCCAACTCTCTCCATCCCCACTCGCCGCCGCTCCCCACCACCCACCGCCCCCGCCGCCGCCTCTCCTCCCCTCATCTCCCTCTCCTCCGAAAACCCTAACAACTTCCTCACTGACCTCCTCTCCGCCTCTTCCCAACTCGGTTTCTTCCAACTCACCAACTCCTCCATACCTTCCCAACTCGCTCGCTCCGTCGAGTCCGAGTCCCTTTCTCTCTTCAACCTCCCGCGCGAACAAAAACAACTCTACTTCCCCCAAAAATGGCCACTGGGCTTCCACTGCGAAGAAGACGAAGACGACAACGGAATGAGTGATTCGTTCTGTTTCGACTCATCTTGTTCTATCGAGTCAACCGAGTTGTCCTTAGCTTCACTCGGTGAGTTCACTCGTGAAATGGAGAAGGTGGGGCTAGTGGTTGTCGAAGCGCTATCGTGCGCAGTGGGATTTGAAAACCCGGTCCGAGAGGACCCCACTCGGGTCTGCTCGTTCATGTGGATATCCGAAGGCTCTCCGGGCAACGTACCGGGTAGGTTTTACCCGTGTGTAGTCGGGTTGCATTACCAAATTAGGTGGCAGAGATGCTCATTGTTGGCGGATTCGGGTTGGGTTTCAGTCACGCCCGAGATCGACTCGATCTTGGTCACTCTTGGCGACGTTGCTCAGGTGTGGAGCAATGGCAAGATAAAGAAAGTGAGAGGAAGACCTGTTCCAATTTATGGGGATGATAGCAACAATGGACGCTGCATATCCATGTCGCTTCTTGTCACTCTTCCAGTAGAAAGCACCGTCTCTCCCCTCATTCCAAGGTTGATCTCTGTTTATGGCGAAGATTACCCAATCAACAAAGATGGTGGTGGAAGTTCTACAGATAGAGGCGGAGAGGGTAGGGCGTTCAGCTCCTTTTCTTTTGAGGACTATGCATGGAGAGTCTACCATGAACGTCTCCATCTTAAGGACTCGCTTGATCAATACCGCATCTAA

>Cha09g017700, *CsGA2ox9*

ATGGTGGTGGCCACTCCAACCCAAATTCACATTGAAAAAATCAGAGAAGTTGAGCTTCCAATCATCGACCTCTCGGCAGAGAGATCAGAGGTGTCAAAGCTCATTGTAAAAGCTTGTTCAGAGTTTGGTTTCTTCAAGGTGATCAATCATGGTGTTCCTGAAGATGTCATTACAAGGATGGAGGAAGAAAGCTATGGATTTTTTGCTAAACCAGGCTGCGAAAAACAGCGGGCCGGGCCAGTTAACCCGTATGGTTATGGAAGCAAGAATATTGGCTTCAATGGAGATGTTGGAGAGGCAATGACAAATGGAAGGTTTGTGAGTGTGAGACATAGAGTCATGACCAACCCATACAAGTCCAGAATGTCAATGGCTTATTTTGGTGCACCACCACTCCATGCCACCATTGCCGCAATACCGGAGTTGGTCACGCAGTGCAGGCCATCTCTCTACTGGCCCTTCACTTGGTCTGAATACAAGAAATCTGCGTATTCTCTACGCCTCGGAGAGAGTCGTCTCAATCTCTTTAGAATACTGGAAGATGATGAAATTGCCTTACCAGAAGCATAG

>Cha11g000330, *CsGA2ox11*

ATGGTTGTTCCATCTCCAACTCCTTCGTTACGAACCAAAAAGACGAGGGCTGTGGGAATTCCCATCATTGATCTCTCTCTGGAGAGGTCTGTGTTGAGCCAGAAAATTGTACAAGCTTGTGAAGAATATGGTTTCTTCAAGGTGGTGAACCATGGTGTTCCCAAGGAGATCATTTCCAGTGTGGAAAAAGAAGCCTCTGACTTTTTTGCCAAAAGGAGTTCGGAGAAGCAAAGAGCAGGGCCACCAAGTCCTTTTGGTTATGGCTGCAAAAATATTGGCTTCAATGGCGATAAGGGTGAGCTTGAGTATCTTCTCCTTCAAACCGACCCTGCTTCCATTTCTGAGAGATCTAAAGCTATCTCCAATAACCCATCAAAGTTCAGCTGTGTTGTGAATGATTATATACAAGCCGTTAGGGACCTCTCATGTGAAATTTTAAATCTAATAGCTGAAGGCTTGTGGGTTCCAGACAATTCTGTTTTCAGTAAGCTCATCAGAGATGCCCATAATGACTCTTGTTTTAGGCTCAATTACTACCCTCCTCTCAAGCATTTTAGGGAGCTAGAATTATCTCCAAAACTTAATCCAGACACGCGTATGGGATTTGGAGAGCATACTGACCCTCAAATCTTGACTATCTTGCGATCAAATGATGTTGGAGGCCTTCAAATTTGTTTAGATGATGGGTTGTGGGTTCCAGTCCTTCCTGATCCTAATGTATTTTGTGTGTTCGTGGGTGATGCTTTAAAGGCTATGACAAATGGAAGGTTTGTGAGCGCAAGCCACAGAGTACTTGTGACAAACACAATCAAACCCAGAATGTCAACAATATATTTTGGGGCTCCACCCCTTGATGCCTACATCTCTCCGATTCCAGAGTTGGTATCACCCCAAAAACCAAGTCTCTACAAGCATTTCACTTGGAGTGAATTCAAAAAACTTTCTATTCTCTACGTTTGGGAGACTGTCGTCTCGACCTCTTCAAGACACTTATGTGCAGTGATAAAACTTCGTCATGTTAACGAATAA

>Cha14g001160, *CsGA3ox14*

ATGACTACTTTCTCAGAGGTTTATACAAACAATCCTCTCCAACTTCACCATATCATCCCCCTCGACTTCAACTCTCTCCAAGCTGTCCCAGACTCCCACATATGGCCTAAATCCGATGATGATGTCCCAACATCCGATGAATATTTGTCGATTCCGATCGTGGACCTCATGGATCCCGATGTCGTGGACCGTGTCAGCCATGCATGTCAGACATGGGGGATCTTTCAAGTCACCAACCATGGACTTTCTCCGAGTCTTTTGGAGGGTGTCGAGGAGGAGACGTGGCGGCTATTCTCTCTCCCGGTCCCGGAGAAGATGAAGGTGCTCCGATTGCCTGACGGAGCCACCGGTTATGGCACTGCTCGGATAGCTCCTTTCTACTCCAAGTCCATGTGGCATGAAGGGTTCACCATCATAGGAGGCTCTTTTGTTGAGCATGCTAAGGTTCTTTGGCCTAATGACTATAAACGCTTTTGTGACGTAATCGATGAGTGCCAGAAGAGCATGAAAACCCTAGCACACAAACTCTTGCGCATCATCCTCAAGTCTCTCAACCTCTCCGAAGAACAAGAATCAAACTGGGCAACAATGATTCATCAATCCGAAACCAATGCGTTACAACTAAATTCCTACCCTACATGCCCGGACCCGAACCAAGCCATCGGCCTACCACCCCACACCGACTCACTCCTCCTCACCATCCTCCACCATTCCAACACCTCCGGCCTCCAAATCTTCCGTGATGGCGGCTTCGGATGGGTACCGGTTTCTTCTTTCCCCGGTGCGCTCACCGTCAATGTCGGCGACCTCCTCCACATCTTGTCCAATGGAAAGTTTCCGACGGTTTATCACCGGGTGGTCGTGAGTGGGACCTGCCACCGGGTCTCTATGGGGTATTTTTATGGTCTGCCGGTTGACTCGACGGTTGAACCACTTTCTAAGGTTGAGTTTCCGATGTATAAGTCTTTGACTGTGAGAGAGTATATTGGGATTAAGAACGAGTGCCATGAAAAGGCACTTTCTTTGATTAGAATCTAG

>Cha15g009060, *CsGA3ox15*

ATGCCTTCGAGAGTCTCCGATGCCTTTAGAACCCACACTGTTCACTCTCCTTATAAACATCTAGACTTCAATTCACTAGAAGAATTGCCCGAGTCCCATGCATGGACATCACTCGAGAAGTATTCTTTGAGTGACTCTGTTGAGACAATAACGGGTACTAAGGAGTCAGTACCCGTTATTGATCTCAATGATCCAAATGCAATGGAACTCGTGGGCCATGCATGCAGGACTTGGGGTGTGTTCCAAGTCACGAACCATGGTATCAACACGAGCGTTCTCAATCGCGTTGAATCCGCAGGGAAGAGCCTTTTCTCTCTCCCTATCCAGCAAAAGCTCAAAGCCGCTCGATCGCCAGAGGGTGTTTCCGGTTACGGTGTTGCTCGGATTTCTCCCTTCTTTCCCAAACTCATGTGGTCTGAAGGGTTCACAATTTTCGGATCACCACTAGAACATGCTTGCCAACTTTGGCCCCAAGATTACAGCAATTTCTGTGATGTGATTGAAGAATATGAGAAAGAAATGAAGATCCTAGCAAGGAGGTTAATGTGGCTAATGCTTGGATCATTGGACATATCCAAAGAAGATGTCAAATGGGCCGGCCCAAAAGGAGATTTCAAAGGGGCTAATGCAGCCGTACAGCTGAATTCTTACCCGGCATGTCCGGAGCCAGACCGGGCCATGGGTCTAGCTGCCCATACCGACTCAACCCTGCTCACCATTCTCTACCAAAACAACACAAGTGGTTTAGAGGTCCACCGAGACGGATATGGGTGGGTCACGGTTCCACCCATACCTGATACTCTAGTGGTCAACATAGGGGACCTCCTTCACATACTATCAAACGGGTTGTACCCGAGTGTGCTCCACCGGGCTATGGTGAACCGGACCCGACACCGCCTCTCGGTTGCCTACCTCTATGGCCCACCACCCAATGTTCAAATCTCACCACTCTCAAAACTGGTAGACCTGACCCACCCGCCTCTCTACCGACCCGTGACTTGGACTGAGTACCTCGGCACCAAAGCAAAGTATTTTGACAAAGCACTTTCATCGAGTTCGCATCTTCTCTCTTCTTCACAACAAAATTCAAACACACCATCATCAGAAACAAAGACCGCCAAGCTCGAATGCCTCGCCGGAGTCTCAACGAATGTTTCTAGGGTTCCGGTGTACCTCCTGTTGGTAGGCAGAGTTGATAATCAACTCCTTTGCTCAAAGCAAGCAATTGCATGGTTTCGAAAGAGCGAGAATTGCGAACGAAGCATGTGGCTCGTAAGCCCTAGACGAAAGACAACAATGCAGGTGGGTGGGTTTATAGATCCATGA

>Cha04g021780, *CsGA20ox4.4*

ATGAGTTGCTTACAATCATGGCCGGAGCCAATCGTCCGAGTCCAATCTCTCTCCGACAGCGGCATGACCGTAATCCCCGACCGCTACATTAAACGGCTCTCCGAACCGCCATCTCCTGTAGAATCGCCACCAGAGACCGAGGTCAACATCCCGGTCATTGACCTCTCCAACATCTTCTCCGGCGATGACTCTCTCCGCGATGAAACTTTAAGCCTAATTTCAAGCGCGTGCCGCGAGTGGGGATTCTTTCAGGTGGTGAACCACGGCGTGAGCCACGAGTTGATGAGCCGGACTCGGGAGGTGTGGCGCGAGTTCTTTCACTTGCCCGTGGAGGAGAAGATAGCATATGCGAACTCTCCGGCGACTTACGAGGGGTACGGAAGCCGGCTCGGAGTCGAGAAAGGGATGAAGCTGGATTGGAGTGACTATTTTTTTCTTCATTTTCTTCCCAAGTCGCTGAGGGACCAGAAGAAGTGGCCGATGTTTCCTCCGTCGTGCAGGGAATTGGTTGCACAATATGGGGAAGAATTGGTGAGGCTATGTGGAATATTGATGAAAGTGTTTTCAATAAACCTTGGATTGGAAGAAGATTGTCTTCAAAAAGCCTTTGGAGGAGAGGACATTGGTGCTTGTTTGAGGGTGAATTTCTATCCAAAATGTCCACAACCAGACCTGGCTCTGGGTCTCTCCCCACACTCTGACCCCGGCGGCATGACCCTTCTCCTCCCCGATGAAAATGTCTCTGGCCTCCAAGTCCGCCGTGGCCATGGCTGGGTCACAGTCAAGCCTGCCCCTAATGCTTTCATTGTCAATATTGGCGATCAAATTCAGGTTCTAAGCAATGCAAATTACAAGAGTGTGGAGCATAGGGTGATTGTGAATTCAGCCAAAGAGAGAGTATCACTTGCCTTCTTCTACAATCCCAATGGGAAAATTCCGATCAAACCGGCCGAAAAACTCGTCTCCGAAGACCGACCGGCGCTGTATCCGGCAATGACATTTAATGAGTACAGATTGTTCATTAGGACAAGGGGTCCTTGTGGCAAATCTCAAGTTGAATCACTAAAATCCCAACATTAA

>Cha03g006460, *CsGA3ox3.2*

ATGATCTCTCTCTATTCCACTATGCCTTCAAGAGTCCCTGATGCCTCTCACCATAAACATTTAGACTTCAATTCAGTAAAAGAACTACCAGAGTCTCATGCATGGACATCACTACTAGGCGATTACCCCTCTGGACCCGACTCATGTGGTGTCGGGTCAGTACCGATTATTGATCTCGACGACCCGAATGCCCAAAAACTTGTAGGTCATGCATGCAGTACTTGGGGTGTGTTCCAAGTCACCAACCATGGCGTCAAAAATAGCCTTCTCAACAGCATTGAAGCCGCTGGGAAGAGCCTTTTCTCTCTCCCGATTCAGCAGAAGCTAAAAGCAGCTCGCTCACCTGATGGTGTCTCTGGCTATGGCGTGGCTCGCATTTCTTCTTTCTTTCCCAAACTCATGTGGTCTGAAGGGTTCACTATTTTTGGATCACCACTCGAACATGCTCGCCAACTTTGGCCCCAAGATCATAATCAATTCTGGTATGAGTAA

>ChaUn1649.3, *CsGA2ox.un1*

ATGGTGGTTCTCGCTAAACCAGCAATTGAACAGTTATCAGCCATCAAAACCTGCAAGCTCACCACCACATTTTTCTCTGGAATTCCCATCATAGACCTATCGAAACCCGACTCAAAGAACCTCCTCGTTAAGGCCTGCGAAGAGTTTGGATTCTTCAAAGTGATCAACCATGGCGTCCCAACTGAATGCATCACCAAACTAGAATCCGAAGCTGTCAGTTTCTTTTCCTTACCACTTTCCGAGAAAGAAAAAGCAGGGCCTCCCAATCCATTCGGGTATGGAAACAAGAGCATTGGATCCAACGGCGATGTGGGTTGGGTCGAATATCTCCTCCTCACAACCAACCCTGAATTCAATTACCAGGGATTTGAATCAATCTACGGCAAAACCCCAGAAACCATTCGTTGTGCTGTGGATGATTATGTGTTGGCAGTGAAGAGAATGGCCTGTGAAGTTCTCGAGTTATTGGCTGATGGGTTGAAGATTCAGCAGAGGAATGTGTTCAGTAAGCTGTTAATGGATGAACAGAGTGACTCTGTTTTTAGGCTGAATCACTACCCTCCATACCCAGAGCTGCAAGAAATGAATGGTAGTAATTTTATTGGATTTGGAGAGCACACTGACCCTCAGATCATCTCTGTTCTGAGATCCAACAACACATCTGGACTTCAAATCTCTTTGAAAGATGGCAACTGGATTTCTGTCCCTCCTGACCAGAATTCCTTCTTCATTAATGTTGGTGATTCTTTGCAGGTGATGACCAATGGGAGGTTTAAAAGTGTAAGGCATAGAGTTATAGCGAACAGTTTGAAATCAAGGGTTTCAATGATATATTTTTGGAGGACCACCATTGAGTGA

>Cha03g006450, *CsGA3ox3.3*

ATGCAGCAACTTGCAGGGAGGTTGATGTGGCTAATGCTTGGATCATTGGGCATAACCAAGGAGGATATCAAATGGGCCGGCCCAAAAGCAGATTTCAAAGGGGCTAGTGCTGCCATACAGCTAAATTCTTACCCGGCATGTCCTGACCCGGATCGGGCCATGGGTTTAGCTGCTCACACGGACTCGACCCTACTCACCATTCTCCACCAGAACAACACCAGCGGCTTGCAAGTCCTCCGGGAGGGAGCCGGGTGGGTCACGGTTCCTCCACAACCCGGTACTCTCGTGGTCAACATAGGGGACCTCCTACACATACTATCAAACGGGTTGTACCCGAGTGTCCTCCACCGGGCTATGGTGAACCGGACCCAACATCGGCTCTCCATAGCCTACCTCTATGGCCCACCCACTAATGTTCAAATCTCACCACTCTCAAAACTAGTAGACCCAAGCCACCCTCCTCTCTACCGGCCGGTCACTTGGACCGAGTACCTTGGCACCAAAGCAAAACATTTTAACAAGGCACTTTCATCGGTCCGGCTATGCATTCCGCTAAATGGATTGGTTGATCAAAATGACAACAAGAGTAAAACAAGCCCACATGCAAAGAGCAAGAGTCAGAATTTTTGGTCCACAAGGGGGGAGATTAAAGTAAAGATGTTGATAGGCTTACATTTTCAGTATCACCTCGAGATTGTGGTGTTGGAGAGGGTGGTTGGGCAGGGCTTTGCTTCAGTCCAAGTCAATGGTCCATGGTTGATGTGGCAGAGCTACGGCAAAAGCTGCAAACTATGGAGACCTAACAAAAAGAACTTGAACTTCTTCAAAGACAAAAGGCTGCCTCTGAACAAGCTGCCTTGA

>ChaUn6953.7, *CsGA20ox.un3*

ATGGACTCAAGTTCCCCAACTCTTCTTCTATGTCCTCCTCCAATAGAGATCAAAGATGAGAGAGAGAAAAAGGTTCTATATTTTGACACATCTTTGCTACAAAAGCAACCAACCTTGCCAACAGAGTTCATTTGGCCACACAAAGACTTATTACACACTCAAGAAGAGCTGAGTGAACCCCTAGTAGATCTAGAGGGTTTCTTTAAAGGAGATGAGGCTGCAACCTCATGTGCTGCCAAGCTTGTTAAGACCGCCTGCCTCAACCATGGCTTCTTCCAAGTTACTAACCATGGCGTCGACAAAGGCCTCATTCGAGCTGCCCATGATCACATGGATGCTTTTTTCAAGCTGCCCATTAACAAGAAGCTTAGAGCTCGCAGAAAACCGGGCAGCGTATGTGGGTATTCGGGTGCCCATGCTGACCGGTTCAAGTCTAAACTGCCATGGAAGGAGACCTTGTCTTTTGGCTACCATGGCAATGGCCCTGTGGTGGTGGATTACTTCAAATCTATCCTGGGAGATGATTTTAAAGAGACAGGGTTGATTTTTGAAAGATACTGTGAAGAAATGAGGCGATTATCACTAGCGCTTATGGAGCTCTTAGCGATTAGCCTGGGAATCGAGCGATTACACTACAGGAAATTCTTTGCAGATGGTAGTTCAATAATGAGGTGCAACTATTATCCACCTTGCAAAGAACCTGGCCTCACCCTCGGCACAGGACCTCACTGCGATCCAACGTCGTTAACAATTCTTCATCAGGACGAAGTTGGAGGCCTCGAAGTTTTTGCTAATAACAAATGGCAGGGTGTTCGACCTCGCCCAGATGCCCTTGTCATTAACATTGGTGACACATTCATGGCATTATGTAATGGAAGATACAAGAGTTGCATGCATAGGGCAGTGGTGAACAAGGAGAGAGAGAGAAGATCACTGGCATACTTTGTGTGCCCAAGAGAAGACAAAGTG

>ChaUn6565.4, *CsGA2ox.un4*

ATGGCACCGGCAATTGAAGAAGAACATGTTGATTCTCTCTCATCCAACGGTCAGAAACTCTGCATCAAAAACTTCATTTGGTCTGACTCAGAATGGCCCCAAATCAACTACGACCACTTCGACGACCGCGATGACATCCCTCTCATAAGTCTCCAGAAAGATCAAAACTGCGAGAAAGTATGTCGTGACATGGTAGCGGCTAGCAACAAATGGGGGTTCTTCAAGCTGGTCGACCATGGCGTGCCATCGGAGATAGTAGAGAACATGAAGGCTCGGTTGAACGAGATGTTCGACCTACCCATGGAGGAGAAGCTGAAGGGAGCGAGATCGGCGACTATGCCACTGGGTTACTGTACTACTAATCCAGATTATGGGAAGAACTTGCCGTGGGCTGAGATATTGCAATTGCTTCAATCTCCTCAACAGGTTTTGGGTTTTGCTAAGAAGGTGTTTGGGGATCAACATCAACCATTTAGTGAGGCAATGGTGGAGTACATGGAAGCACTAGACAAGCTAGGAATGACAATAATGGAGATGTTGGCTAATGGGTTAGGGCTTCAAAGTGATTTCTTCACAAAGAACTTTGAAGAAAAGGACTCAACCATGATCAGGATTAACAAATACCCTCCTTGCCCCTTACCTGAAAAATGTCTAGGTGTGGGCAGCCACACTGACCCTCACACCTTGACAATATTGTTGGCAGACAACGTTGGTGGTCTTCAAGTTCTCAAGGATGACAATCAGTGGGTTGGAATCCGACCCATCCCTAACTCCTTTGTCATCAACATTGGTGACACCCTTGAGGCATGGACAAATGGGAGGCTAAAGAGTGTGATACACAGAGCAGTGGTGAACAAAGAGAAGAGCAGACTATCAATTGCATATTTCCTAAGCCCTGCACTTAGTACCATCATAGACAGTCCTCCAGAGCTCTTGGAGGACCCTAACACAGACCCCCCTATAAATAGAAAGTACGTCCCATTCACTTGGGCTGACTTTAGGAAGGAGCTTCTCATTCAAAAGAGGGTTATTGGCAAAACTGCACTTAACAGATATCTCATTTCTCCTTGA

>ChaUn13888.1, *CsGA2ox.un6*

ATGGTTGTGTCATCTCCGACTCCATTTCGGACCAAGAAGGCAAGGGCTGTGGGAATTCCCATTATTGACCTCTCTCTTGAGAGGTCTGTGTTGTCTGAGAAAATTGTGCAGGCATGTCAAGACTATGGTTTCTTCAAGGTGGTCAACCATGGTGTTCCCATGGAGATCATTGCAAGAATGGAGAAGGAAGGGAGTGATTTTTTCGCAAAACCCGCTTCGGAGAAGCAACAACAAGCGGGGGACCTGCCTAGTCCATTTGGTTATGGCTGCAAAAATATTGGCTTCAACGGTGATGTGGGTGAGCTTGAGTATCTTCTTCTTCACACAAACCATCTCTCCATTTCTGACATATCCAAAGCTATCTCCTCTATTAACCCAACAAAGTTCAGTTGTGCTGTGAATGATTATATACAAGCAGTTAGGAGCTTGACATGCGAGATGCTAGATCTAGTGGCTGAAGGTTTATTTGTTCCAGACAAATCCGTCTTCAGTAGGCTTATCGGAGACGTCCATAGTGATTCATGTTTCAGGCTCAATCACTACCCTCCTATGGAAAACTCTAGGGAATGGGATCCATCTCCAAAGCTTAATGCAAACACTCGTATGGGGTTTGGAGAGCATTCTGACCCTCAAATCTTGACCATCTTGCGATCAAACGATGTTGAAGGCCTTCAAATTTGTTTACATGATGGGTTATGGGTCCCTGTCACTCCTGACCCCACTGAATTTTGCGTGTTTGTAGGCTATGACAAATGGAAAGTTTGTGAGCGCAAGACACAGAGTAATAGTAGCAAACTCAGTGAAACCCAGAATGTCAATGATATATTTTGGGGCTCCACCCCTTAA

>ChaUn8759.2, *CsGA2ox.un8*

ATGGTGGAGAACTGGTGCCCATCGAACCAAGCAATGGTGAAGATTGATAAAATTCCAGTGCATCAAAGCCAGTCTTCAAGCATGGACTCATCAGAGCCACCATTTCAAGAGGCTTACAAAACCCTCTTCCTCAACTACACCGAAGCAGAATCAAACCACGCCAACGAGCTCATCATCGTCAAAGAATGTGAATTACCGGTGATTGATTTGAGCCGGTTAGAGCTCGGAGAATCAGAGAGAGAAGAGTGCAAGAGAGAGATAGCTAAAGCTTCACAAGAATGGGGGTTCTTCCAAGTTGTGAACCATGGGATTTCTACAGAGATTTTGGAGAGAATGAGATGTGAGCAAGTGAAGGTGTTCAAGAGGCCTTTTAGGGAGAAGATAAGAGAAGGGTATATGAATTTCTCGGCGGGTAGTTACCGGTGGGGAACACCATGGGCGACTTGCCTGAGGCAGATATCTTGGTCTGAAGCATTTCATGTTCCTTTGGACGATATTTGTGGCTTGGGTGGTCTCACCACTCTCAGCTCAACAATGGAACAATTTGCCACAACAGTTTCAGACTTGGCACAAAAGCTTGCTGAAATATTGGCAGAGAAAATGGGTCACAAAGCCACATTTTTCAAAGAAAATTGTGTGCCCAGTACTTGCTATCTCCGAATGAACAGATACCCACCATGTCCAATATCTTCTGAGATCTTTGGATTAATACCACACACAGACAGTGACTTCCTCACTGTATTGCATCAAGATCAAATTGGAGGATTGCAGCTAGTGAAAGATGGGAAATGGATTGCAGTTAAACCTAATCCTGATGCCCTCATCATTAACATTGGTGACTTGTTTCAGGTATGGAGCAATGGTGTTTATAGGAGTGTTGAACACCGGGTTGTGGCCAACAAGAAAATGGAGAGATTCTCTACTGCATACTTCTTTTGTCCATCCTATGACACTCTGATGCAGAGTTGTGTTGAGCCTTCAATCTATAGAAGGTTTAGCTTTAGAGAATTCAGACAACAAGTCCAAGAGGATGTCAAGAGATTTGGCCACAAAATAGGGCTTCCTAGGTTTCTTGTTTGA

>Cha10g004160, *CsGA20ox10*

ATGAACTCCTTACAATCATGGCCCGAACCAATCATCCGAGTTCAATCCCTCTCCGACAGCGGCCTAACCTTTATCCCAGACCAGTACGTAAAACCACTCGCCGACAGGCCATCTCTAACCGAACCACCACCACCACCAGCCGAAATCAACATCCCGGTCATTGACCTCTCCCAACTCTTCTCCCCCGACCGCTCAATCCGATCCGCTACAGCGCGTGTCATCTCACGAGCGTGCAGCGAGTGGGGGTTCTTTCAAGTGGTGAACCACGGCGTGAGCCACGAGCTGATGAAGCGGATCCGAGAGGTGTGGCGCGAGTTTTTTGAACTTCCCTCGGAGGAGAAACAAGCCTACGCGAACTCACCGGCGACTTACGAGGGGTACGGAAGCCGGCTCGGAGTGGAGAAAGGAATGAAGCTGGATTGGAGTGATTACTTCTTTCTTCATTACCTTCCGGAGTCGCTGCGTGATCAGAATAAGTGGCCGAGTCTTCCTCTCTCGTGCAGGGAATTGGTGGCAGAGTATGGAAATGAACTGATTAAACTGTGTGGAAAATTGATGAAGGTTTTCTCAGTGAACCTGGGATTGGAAGAGGACTACCTTGGAAAAGCTTTTGGAGGAGAAGAAGTAGCTGCTTGTATGAGGGTGAATTTCTATCCAAAGTGTCCACAACCTGACCTCACATTGGGTCTCTCCTCACACTCGGACCCCGGCGGCATGACCCTTCTCCACTCCGATGATCATGTCGCAGGCCTCCAAGTCCGGCGAGGTGATGACTGGGTCACTGTTAAACCTGTTCCTAATGCCTTCATTGTCAACATTGGTGATCAAATTCAGATTCTAAGCAATGCAACATACAAGAGTGTGGAGCATAGGGTGATTGTGAATTCAGCCAAAGAGAGACTATCACTTGCCTTCTTCTACAACCCTAATGGCAACATTCTCATTGAACCCGCCAGTGAGCTCGTGACGGAGGACTCACCGGCTTTGTATCCGGCAATGACATTCAACAAGTACAGATTGTTCATCAGAACAAAGGGTCCTCGTGGCAAGTCACAAGTTGAAGCACTCAAATCACCCCATTAA

>ChaUn12153.4, *CsGA2ox.un*

ATGAAAGAGCGAGAGGAGCGCAAGAGACGTTACAATGAGATGTTGAAAGAACGAGAGGAGCGCATTAAATTAGCCAATGAGATGTTGAAAGAGCGGGAGGAGGGACAAAAAGCGAGGAAGGAGACAAATACAACGGTCGAGCATCTTAATAACGTAGTCGCCCAAACTGTCACAACTCTTGGGAATCTAAGTTGGGGCATTCCGACGTTTGCTTTTGCCATAGCCACAGAGAGGAGTCCTAGTTCCCTAAGCATTAACTGGGTCGAAGGTTTTAATGTTCCTCTGAGTCAACTCTCTGTGCTTCAAGCTGATGGAGATCCAATTCTTGATACTTTCAGACATTTGTTGGATGAGTATGGAAGGCATCAATCCAGAGTAGCCACCACAATTTTCAAAGCCATGGCAGAGAACCTCAACCTTTTAGGTCAAGAAAAATGCAAGTCCTATATCTCTCCATCTACTAGCTTTATACGTGTCTATCGCTACCCACGTTGCTCTGAGTCCAACCACAACAACCGAGCCTCTGCTTGGGGCATGGACGCCCACACAGACAGCTCAGTGATCTCCATATTGAACCATCATGATCAAGTTGGAGGACTTGAAGTCTACAAAGACCACAAATGGCTCGACATTAAACCTATTCCTAATACACTTGTCGTTAACCTTGGAGATATGACCCAGGCTATGAGCGATGATGAATACAAGAGTGTGAAACATAGAGTGAAGGCAAACAAGTACGACGACCGAATATCAATTGGCTACTTTGTGTTCCCAGCTCAAAATGGTGTGATTCAGAGCTCCAAGTACAAGCCTTTTACTTATTCTGATTTTCAAGCCCAAGTTCAAAAAGACCTGATGACCATGGGCTTCAAAGTTGGGCTTGACCGCTTCAAGCTTAATCAGGCCTCTTAA

>Cha04g016560, *CsGA2ox4.2*

ATGGAAAAGCAAAAGCAGCAGAGACGTACATCCACTTTTTTTGACTTCTGCAAAGCAGAGAAGGGCATCTTATTATGTACTGATGTTGCTGCTCGCGGTCTTGATATTCCTGCAGTGGTGGATGACAAGTACCAATACTCAACTGAGGACAAAGATAATCAGGTTCACGGGTGGATATGCTTTGACCCGCCTGTGGGGTTCTGGCAAATCACACCCAGCAATGAGTTCCGAACCGGTGGACCCTTCAAACAAGACCTAACCTCCCATGTCAACCCAACAACCCTTGCTGTTGAGCAAAAAAGGAAAGAAGCGCTTTCCAGAACAGGCGAAGAGAGTCAATCAGCTTTAGAGAGTTCACTACAAGATGTTGTATCATGTTATAGTTTCATGGATCTTTGGCCTTGCTCTTCTAAGGATATGGATCATTTGGCACGGCAAGAGGTGATGACCAATGGGAGGTTTAAAAGTGTAAGGCATAGAGTTATAGCGAACAGTTTGAAATCAAGGGTTTCAATGATATATTTTGGAGGACCACCATTGAGTGAAAAGATAGCCCCATTGCCTTCACTCATGGAAGGAGAAGACAGCTTGTACAAAGAGTTTACATGGTTTGAATACAAAAAATCTGCATACAAGTCTAGACTTGCTGATAATAGGTTGGGCCTGTTTGAGAAAATTGCAGCCTCATAA

>Cha05g016340, *CsGA3ox5.5*

ATGGAAAACCTAGCCGTGAAACATAGGGTGATGATGAGTGGTGAGAAAGAGAGGTATTCATGTGGCTTCTTTACAATACCAAAGGATGGAGTGGGCATTAAAGTACCTCAGGAGCTTGTGGACAAAAACCATCCTCTCCTCTATCTCCCATTCATTTATTCTGAATACCTTCAAAAACACCTTACTGACCTTAGCTTCAATGATATGAACGTGATGTTGGAACTGAACTTGCTACTATTGAGAATGATCATGGAGAGTTATGGGGTTGGAGAGTACTACAAGTCAAAGGCATTTGGTGACAACACAGGAAACAATTTTAGAGCGATGAAGTACAAAGTTCCTAAAAGCAAGAATGAAGAGGGAATAGGGCTTGGGCCTCATGTTGACAAAACCATCCTTACCTTTTTATGTGATAATTCAGTGCGAGGTCTTGAGGCCCTCACTCAAGATGGAATTTGGATTTCACTTCACATTCCCAAGGGTGCCTTGTTGGTCATGGTTGGTGATGCCCTTGAGGTTTTGAGCAATGGAAGGCTTCAAGCAGCGAAACACAGGGTCATAATGAGTGGTGAGAAAGAGAGATATTCATGTGCTTTCTATACAATACCAAAGGATGGAGTGGGCATTGAAGTACCTCAAGAGCTTGTTGACGAAGACCATCCTCTCCTCTATCGCCCCTTTGTTTATTCTGAATACCTTGAAAAACACCTTACCAACACTAATTTCGATGCACTCCAAGTTCATGCCGGTATTTGA

>Cha08g009430, *CsGA3ox8.2*

ATGGGTGAGGCTGCTGAAATGATCCCCTCAATTTCTCTTACAAATGAGGTATTGAGTTTGGATAGACACAGCGAAGAATGGAAAGCGATGACTGGAAAGGTGAGAGAGGCCTGCGAAGCATATGGTTGCTTTATGCTTGATTATGAGAAGATCCCATTAGAGCTTCAACAAATACTGTTCAAGACCACCGCCGAGATGTTCGATCTCCCAGTGGAGACCAAGTTGCAGTACAAAAAACCTACTCCTAAAGATGGTGGTTACGTTCTCAGCAACCCGTTTATCCCTCTCTACGAGAGCTTTGGAGTCTATGGCGAAGAAGAGGTTCGAGCTTTCGAGAGGCTTATGTGGCCTCAAGGCAATCCTCAATTCTGTGAGGTTATCAACTCCATAACTGCAAAGGTGCATGAACTGAACCTCCTATTACTGAGAATGATTTTCGAGAGCTTTGGGATAGAAGAGTACTACAAGGAGAAAGCACTAGATGAGAACATTATTGGGTATTTACGTTCCATGAAGTACAGACTCCCTAAAGAGGATGAAGACCCTCTTGGTCTCGCAATTCACGTTGATAAAACCATCATAACCATTTTATGTCAAAATTCAGTGCAAGGTCTTGAGGTTCTCACTAAAGAAGGAAAATGGATTAAACTTAGCATTCCTAAGGGAACCTTGGTAGTCATTGTCGGCGATGGGCTCCAGGCTTGGACCAATGGAAGAGTTGAAGCAGTGCAACACAGGGTGGTGATGAGCGGTGACAAAGAGAGGCTTTCGTGCGGATTCTTTTCTATTCCAAAGGATGGAGTGAGTGTTGAGATACATCCACAGCTTGTAGACGAAGAACATCCTCTGCGCTACCGATCATTCGTTTATTCTGATTATCTTGCCAAATACCTTGCCAATACTAGGTTGGATGCGCTCGAGCTTTTTGCCGGTATTTGCTGA

>Cha03g005890, *CsGA2ox3.1*

ATGGCAGTTTCGCCAGAATCGTCGGCCGATCAAAATCCAGTCGATTTCCGAGCGCCGCCGCCTTCTCCGATTGCCTCCGGACGCCGATCATCCTTCAAAAACGACGACATTCTCACCGACTTCCTCGAACACTCTCTCCGCGTCCCCGACCTCATCTTACCCGATCGGTTCTTCCCTCGCCAAAAATCGATCCAAAACCCTCCCAAAATCGACTTTCAGTCCCTTAATTCACCTGAAAACGATGACTCCGTCGCCGAAATGCTAGACGCTATCGCTCGATTTGGTTTTTTTCAGATCGTCAATCATGGAATTACGCGCGAACAAATCGCGTCGGTTTCAATGTCCGGTGCCGGAATCTTTAGGTTGTCACCGGAGAAGAAGGCGGTGGTGTCGAGATCGCCGGAGAAACCGTATGGATTTGAGGAGTTCCATGGAGAAGTTGAGAGTGAGATGAGCGAAGAGTTTGTTTGGTGTAGAGACCATGGGTTGAAGTTGGAAATGGAGGGAGTTTGGCCACTTGGATATTCAAAGTTCAGTGAGAACATGGAAATGCTTTTGGTGGAGATAGAGAAGGTAGGTGGAAGAGTAGTATTAGCTATGGAGGAGCATAATGCAAGAAAATCACAATGTGGAAATGGGATCATACGAGGACAAGATTTTGATGGCTCAGTTTGTTGTTTCTACAAACATAGCCGCAATGTTTCTGGTGACCAGTGGGTCGGCTCCTTGAAATGTGATGTGATTAAGATGCTGATCAGAGGATCAGATTACTCTCATGCACTATGTATGCATGTCTGTGAAGGGTCTTCTGAGTTTCATGTTTACTCCAAGAAAGGTTGGGTATCATTCTGCCCTGACACAGATGCTATAGTCATCACAATTGGAGATCAAATACAGGCATGGAGTGGTGGACTGTACAAGCAGGTGATAGGGAGGCCAATATTTAAAGGAGAAAATGAAGAGTGTATCTCAATGGCTTTCCTCTATTCTCCCTCTAAAAACATCAAAAGCTTCAAAACAAACGAGGGGAAGACCATATCACTTGGCCAACAAGCCATAGTGGCCATACTTTTCACACTTGTTTACCATTTTTTGGTTTATATCTACAAATTGTGA

>Cha07g008840, *CsGA2ox7.2*

ATGGCTCATGTACAGAGGACTAGTTTTGATGAAAGGGATGTCGAGCAGGTATTCGGAGTTTACTATGCAGAGTTGGATCCAATTTGGCGCTGTCTTATAGAGGTGATGACCAATGGGAGGTTTAAAAGTGTAAGGCATAGAGTTATAGCGAACAGTTTGAAATCAAGGGTTTCAATGATATATTTTGGAGGACCACCATTGAGTGAAAAGATAGCCCCATTGCCTTCACTCATGGAAGGAGAAGACAGCTTGTACAAAGAGTTTACATGGTTTGAATGCAAAAAATCTGCATACAAGTCTAGACTTGCTGATAATAGGTTGGGCCTGTTTGAGAAAATTGCAGCCTCATAA

**List of sequences of putative GAox proteins in LJ43 and GAox proteins in *Arabidopsis***

>CsGA2ox1.2

MATNNQCNVAVIDLEDFPGQSRKMIEACEELGCFRITNHHQFLPPALSAEMKAVVRSLFDLPVEIKRLNTDVIPGSGYMAPSQINPHYEAFGLYDIASHEAVNAFCSPLDASPHQRETIQMYAEAIHNLAMHIACKLAEGMGLTSFSFEGWPCQFRMNKYNFTPETVDSSGVQIHTDAGFLTILQEDENVGGLEVMDKAGKFVAVEPLPGTLLVNLGDIAKVWSNGRLCNVKHRVVCKEARTRMSIASFLLGPKEAKVEAPVQLVDSSHSRLYVPFFYSDYRKLRISKELHAGEALDLLHTNS

>CsGA2ox1.1

MASSTHHLPNPYGTTAAPPPTPSTQPNPLPSSAPSADVLSRLLHRLPPTLSLPTRLSPPPTTITTTTTTSPPLISLSPQNPTLLTDLLSASCQFGFFHLTNHSISPQLARSAESDSLSLFTLSNDQKQIHFPKNWPLGFDCDDNEDDDGTSSDSFCFDSACSPESTELSLSSLREFTREMEKVGLEVVEALSCAVGFENPVREDPTRVCSLMWISEGSPGNTTVRFYPYVVGLEYQIRCRKYSLLADSGRVSDSPRVDSILVTLGDIAQVWSNGKIKKVRGRPVPSFEDGSNNNNAHCISMSLLVTLPLESMVSPLLPKLTVNNGEEDNQLNKEDDDDNGDTSADTREDGAFKFNSFSFEDYAWRIYHERLLLKDPLARYQI

>CsGA2ox4.1

MPPSTTNTTTHYDSYPPVFRPKIASIHHSAPTSRNPYSDSDPLPVIDLQCLNKNKLAEACRDWGIFRLVNHGVPTKLLSQLHEHAKDLFSLSFETKQRHGFGSPMSYFWGTHGLTPSGVAIERGLNIATNINWVEGFSVPLTLLSNLLAQKKLSHQLQDHGDDGDYPMLDAFRTELDEYGKHQSRLATTIFKAIAENLELGRELSDCYLSTSTGNMRVYRYPQCSEENRAWGIDAHTDSSVLSILYHDEVGGFEVYKDGNWLPVKPIPNTLVLLLGDMMQAMSNDKYKSVKHKVKINKYEDRMSAGYFVFPKENGVIRSSNYKPFCYAEFRAQVQHDLKTVGYKIGLDHFKLH

>CsGA20ox4.3

MEESKATTTKDNNIPLIDLSLLNSPNNSSSADSIASLAAEITEACETWGCFQVINHGVPVEQSRAIESASRQFFDQPKEEKKKVWRYERNPLGYHDSEITMNVRDWKEVFDFTVQNPTLFPASPDPHDLELRELVNQWPQFPPQFSLGLPRRRLNHFFKDQTSIVRFNHYPPCPNHPGQVLGVGQHTDSGALSILAQDDVGGLQVKRKWDGKWVPVKPNPHAYLVIICDIFQVWSNDKYESVVHRATVNSEKERISIVFFSYPAHYTMVEPLEELINDRNPPKYRPYNWGKFSAARRRSNFTKNNVGRIQIDHFKVI

>CsGA2ox5.1

MVVATPTPIRSEKIREVELPIIDLSAERSEVSKLIVKACEELGFFKVINHGVPEDVIARMEEESFLFFSKSGSEKQRAGPANPYGYGSKNIGFNGDTGEVEYLILNTNFLSISQRSKAISNDPIKFSCAVRDYVEAVRIVACEILELMAEGLWVPDASVFSKLIRDVDEDDSLFRLNHYPPLLLTDSFQDSDTSPSSFHHHLHNHKIGFGEHSDPQILTILRSNDVSGLQISPQQGLWFPVSPYPTTAFCVNVGDLLQAMTNGRFVSVKHRALASSYKSRMSMAYFGAPKLHARITTPPELVTQFRPCLYRPFTWAEYKETTYSLRLSDSRLNLFKVQEGDEIV

>CsGA2ox5.2

MGTSSLVPVIDLQDFPSQSQKLIDASQEWGCFRIINHQIPTTLMSDMKSVVRSLLDLPPEIKRRNTDVIAGSGYVAPSKVNPLYEGLGLYDIGSTHAVLDFCSQLDASPQQRDTILRYSQSIHKLAMDIGNKLAQSMGLASDLFMGWPCQLRINKYSFTPETLGSSGVQIHTDSAFLTILQDDENVGGLEVMDKKSGEFVPVDPMPGTLLVNLGDFATSDLIDTFTNVSKVWSNGRFYNVKHRVQCKEATIRLSIALFVLGPKEAPLEAPPELVDSESHPRLYVPINFEDYRKLRLSTGLRAGEALDKLSASNVPF

>CsGA2ox5.3

MKEDSVGQSEKIVEACREWGCFRIINHGVPPELMAEMKVATQSLMELPVEIKRRRYHPVESHGYVSVNKVSPVYESLLACHDSCVPGALDNFLDLMAVSPHLRKTVVKYFEAINEQALDMGRKLGEGMGLKGDLFKDWSSQMRLNKYHFNPEFVGSSALYLHTDPGFLTILQDDEIVGGLEVVHGKTGERVHVDPMPDSLLVNLGDMATIWSNGRLPSVKHRVQCYEVKTRFSIVFLVWGPNDRATEQPPELVDSQHPRLYNPINFADYRLLRLSNNLFTGAFELLRINS

>CsGA2ox5.4

MVVLSKPAIEQYSVIKTSKPTTFSSSIPLIDLSKPDTKTLLVKACEEFGFFKVINHGVPTELINKLESEALKFFSSPLFEKEKTGPPNPFGYGNKNIGTNGDAGWVEYLLLTTNPEFNYQRFVSIFGKNPEMFRCVVNDYVSAVKKMACEVLELLADGLKIQQRNVLSKLLMDEQSDSVFRLNHYPPCREIQDVNGKHLIGFGEHTDPQIISVLRSNNTCGLQISLRDGNWISVPPDQNSFFINVGDSLQVMTNGRFKSVRHRVVANSLKARISMIYFGGPPLSEKIAPLPSLMEGEDSLYKEFTWFEYKTSAYNSRLADNRLGLFEKIAAS

>CsGA3ox6

MMSDESHTSLQLPIIDISLPLQSPSSLSSLSQACKEWGFFLISDHGVSKELYNKVRSLSEYLFSLPVETKTKLGPLSSVKTYTPHCIASAFFESLRVSGPDFAASAQSSADVLFQHHNSEFSETLQEYGSKMTELSKKIVKIVLMTLGDDFENKFNDSEFNNCHGYLRINNYSPPQILEEEEEEEVEGLGKHTDMSCVTIVYQDEMGGLQVRSKDGRWIEMNPCEGTLVVNVGDMLEAWSNGKLRSSEHRVVLKKPANRFSLAFFWCFEDEKVVLAPEAVVGEGNERVYRPFVCSDYLKFRETNEEGKFEKVGFTVKDFAGINAIVESACLIILADATAAPRN

>CsGA2ox7.1

MVILSQSVVEDFSHIMTCKPTSGLFTGIPEIDLSDPDAKILIVEACQEFGFFKVVNHGVPIELITRLEAESVKFFNLPQSEKDKAGPSIPFGYGNKRIGPNGDVGWVEYLLFSTNPEFISQMSLSIFQDNPEIFKSAVNDYISNVRDMVCEVLELIADGLKIEPRNVLSKLLRDEKSDSCFRLNHYPPCPELQAWSGRNLIGFGEHTDPQIMSAVRSNNTSGLEICLKHGTWVSVPPDQYSFFINVGDSLQVMTNGRFRSVRHRVLADNLKPRVSMIYFGGPPLSEKLTPLCSLMKDGEESLYKEFTWHEYKTSAYKTRLGDNRLGFFLQNASQ

>CsGA2ox7.3

MLPTPFHSLLLSCHVMAGQKYGSMVSKHLRRLQMSYVPTWTQMPGDQCVLKLEHWCSFEKAVMESDKFSADRNQPLDMGSSEPPFQDTYKALFDDYVDAESKYEKNDVVIVAECELPMVDLSRLNLGEEEREDCKREIAKASQDWGFFQVVNHGICREILEKMRREQMKVFKRPFCEKINDEFMNFSEGSYRWGTPSATCLKQLSWSEAFHVPLAHISGLGGLTNLSLTMEQFATTVSDLAQKLAEILAEKMGHKSTFFKENCLPNTCYLRMNRYPPCPISSEVFGLMSHTDSDFLTILHQDQIGGLQLVKDGNWIAVKPNQDALIINIGDLFQAWSNGVYKSVQHRVVANKLEERFSTAYFFCPSYDTEIQSCVEPSVYRRFSFREFRQEVQEDVKKFGYKIGLPSFLVQN

>CsGA2ox8.4

MESEPPFQEIYKTLFVNFSEEGTNSESVHEKKPMILDEYEIPLVDLSRLSLCNLEQKEGKRELASAANQWGLFQVVNHGVSCDVLTRLQREQVKLFRQPFQNKALNSLADYYSWGAPNAATFMQFSWSEAFHIPLNNMPVLKEFNSNLSTTIAEFSEVVCELAQRIAEILAENVGCRSTFFAKNCQPSSCYVRMNRYPPCPVSSSKVYGMVPHTDSDFLTILCQDHVGGLQVAKDGKWVSVKPNPEALIVIIGDLFQAWSNGVYKSIEHRVVTNHEVERFSVGYFFCPTIETVIQTCKEPPLYRKFSFREYRGQVQEDVKTAGTKIGLSKFLITNLCLSHPSSGLSHHGLPLFKRNLNDNQMYLAAEETNITPETRKNTE

>CsGA3ox8.3

MGADAAAEMIPSIALTNEVLSLDRESEEWKAVIRKVREACETYGCFFLKYENLTLELQQLLFKTTGEMFDLPLETKLQYKKPTPRQGGYVVSNPFIPLYESFGVYGEEELGKSRPTDSWMLIFLDSEVINSINAKMLELNLQIQKMIFESFGILEYYKEKALDENIIGNLRAMKYRLPKENEEPLGLAIHVDKTIITILCQNSVQGLEILTKDGKWITLDVPKGALVVFVGDGLQAWTNGRLEAVQHRVVMSGDQERLSCGFFAIPEDGVSIEVPPQLVDEEHPLRYRSFVYSEYLAKYLANTRLDALEIYAGV

>CsGA2ox8.1

MASSAHDQLHQLPNSNYGTTAAPPPTPSTQPNPYSSSDLAADVLSRLLHRLPPTLSIPTRRRSPPPTAPAAASPPLISLSSENPNNFLTDLLSASSQLGFFQLTNSSIPSQLARSVESESLSLFNLPREQKQLYFPQKWPLGFHCEEDEDDNGMSDSFCFDSSCSIESTELSLASLGEFTREMEKVGLVVVEALSCAVGFENPVREDPTRVCSFMWISEGSPGNVPGRFYPCVVGLHYQIRWQRCSLLADSGWVSVTPEIDSILVTLGDVAQVWSNGKIKKVRGRPVPIYGDDSNNGRCISMSLLVTLPVESTVSPLIPRLISVYGEDYPINKDGGGSSTDRGGEGRAFSSFSFEDYAWRVYHERLHLKDSLDQYRI

>CsGA2ox9

MVVATPTQIHIEKIREVELPIIDLSAERSEVSKLIVKACSEFGFFKVINHGVPEDVITRMEEESYGFFAKPGCEKQRAGPVNPYGYGSKNIGFNGDVGEAMTNGRFVSVRHRVMTNPYKSRMSMAYFGAPPLHATIAAIPELVTQCRPSLYWPFTWSEYKKSAYSLRLGESRLNLFRILEDDEIALPEA

>CsGA2ox11

MVVPSPTPSLRTKKTRAVGIPIIDLSLERSVLSQKIVQACEEYGFFKVVNHGVPKEIISSVEKEASDFFAKRSSEKQRAGPPSPFGYGCKNIGFNGDKGELEYLLLQTDPASISERSKAISNNPSKFSCVVNDYIQAVRDLSCEILNLIAEGLWVPDNSVFSKLIRDAHNDSCFRLNYYPPLKHFRELELSPKLNPDTRMGFGEHTDPQILTILRSNDVGGLQICLDDGLWVPVLPDPNVFCVFVGDALKAMTNGRFVSASHRVLVTNTIKPRMSTIYFGAPPLDAYISPIPELVSPQKPSLYKHFTWSEFKKLSILYVWETVVSTSSRHLCAVIKLRHVNE

>CsGA3ox14

MTTFSEVYTNNPLQLHHIIPLDFNSLQAVPDSHIWPKSDDDVPTSDEYLSIPIVDLMDPDVVDRVSHACQTWGIFQVTNHGLSPSLLEGVEEETWRLFSLPVPEKMKVLRLPDGATGYGTARIAPFYSKSMWHEGFTIIGGSFVEHAKVLWPNDYKRFCDVIDECQKSMKTLAHKLLRIILKSLNLSEEQESNWATMIHQSETNALQLNSYPTCPDPNQAIGLPPHTDSLLLTILHHSNTSGLQIFRDGGFGWVPVSSFPGALTVNVGDLLHILSNGKFPTVYHRVVVSGTCHRVSMGYFYGLPVDSTVEPLSKVEFPMYKSLTVREYIGIKNECHEKALSLIRI

>CsGA3ox15

MPSRVSDAFRTHTVHSPYKHLDFNSLEELPESHAWTSLEKYSLSDSVETITGTKESVPVIDLNDPNAMELVGHACRTWGVFQVTNHGINTSVLNRVESAGKSLFSLPIQQKLKAARSPEGVSGYGVARISPFFPKLMWSEGFTIFGSPLEHACQLWPQDYSNFCDVIEEYEKEMKILARRLMWLMLGSLDISKEDVKWAGPKGDFKGANAAVQLNSYPACPEPDRAMGLAAHTDSTLLTILYQNNTSGLEVHRDGYGWVTVPPIPDTLVVNIGDLLHILSNGLYPSVLHRAMVNRTRHRLSVAYLYGPPPNVQISPLSKLVDLTHPPLYRPVTWTEYLGTKAKYFDKALSSSSHLLSSSQQNSNTPSSETKTAKLECLAGVSTNVSRVPVYLLLVGRVDNQLLCSKQAIAWFRKSENCERSMWLVSPRRKTTMQVGGFIDP

>CsGA3ox3.2

MISLYSTMPSRVPDASHHKHLDFNSVKELPESHAWTSLLGDYPSGPDSCGVGSVPIIDLDDPNAQKLVGHACSTWGVFQVTNHGVKNSLLNSIEAAGKSLFSLPIQQKLKAARSPDGVSGYGVARISSFFPKLMWSEGFTIFGSPLEHARQLWPQDHNQFWYE

>CsGA20ox4.4

MSCLQSWPEPIVRVQSLSDSGMTVIPDRYIKRLSEPPSPVESPPETEVNIPVIDLSNIFSGDDSLRDETLSLISSACREWGFFQVVNHGVSHELMSRTREVWREFFHLPVEEKIAYANSPATYEGYGSRLGVEKGMKLDWSDYFFLHFLPKSLRDQKKWPMFPPSCRELVAQYGEELVRLCGILMKVFSINLGLEEDCLQKAFGGEDIGACLRVNFYPKCPQPDLALGLSPHSDPGGMTLLLPDENVSGLQVRRGHGWVTVKPAPNAFIVNIGDQIQVLSNANYKSVEHRVIVNSAKERVSLAFFYNPNGKIPIKPAEKLVSEDRPALYPAMTFNEYRLFIRTRGPCGKSQVESLKSQH

>CsGA2ox.un1

MVVLAKPAIEQLSAIKTCKLTTTFFSGIPIIDLSKPDSKNLLVKACEEFGFFKVINHGVPTECITKLESEAVSFFSLPLSEKEKAGPPNPFGYGNKSIGSNGDVGWVEYLLLTTNPEFNYQGFESIYGKTPETIRCAVDDYVLAVKRMACEVLELLADGLKIQQRNVFSKLLMDEQSDSVFRLNHYPPYPELQEMNGSNFIGFGEHTDPQIISVLRSNNTSGLQISLKDGNWISVPPDQNSFFINVGDSLQVMTNGRFKSVRHRVIANSLKSRVSMIYFWRTTIE

>CsGA3ox3.3

MQQLAGRLMWLMLGSLGITKEDIKWAGPKADFKGASAAIQLNSYPACPDPDRAMGLAAHTDSTLLTILHQNNTSGLQVLREGAGWVTVPPQPGTLVVNIGDLLHILSNGLYPSVLHRAMVNRTQHRLSIAYLYGPPTNVQISPLSKLVDPSHPPLYRPVTWTEYLGTKAKHFNKALSSVRLCIPLNGLVDQNDNKSKTSPHAKSKSQNFWSTRGEIKVKMLIGLHFQYHLEIVVLERVVGQGFASVQVNGPWLMWQSYGKSCKLWRPNKKNLNFFKDKRLPLNKLP

>CsGA20ox.un3

MDSSSPTLLLCPPPIEIKDEREKKVLYFDTSLLQKQPTLPTEFIWPHKDLLHTQEELSEPLVDLEGFFKGDEAATSCAAKLVKTACLNHGFFQVTNHGVDKGLIRAAHDHMDAFFKLPINKKLRARRKPGSVCGYSGAHADRFKSKLPWKETLSFGYHGNGPVVVDYFKSILGDDFKETGLIFERYCEEMRRLSLALMELLAISLGIERLHYRKFFADGSSIMRCNYYPPCKEPGLTLGTGPHCDPTSLTILHQDEVGGLEVFANNKWQGVRPRPDALVINIGDTFMALCNGRYKSCMHRAVVNKERERRSLAYFVCPREDKV

>CsGA2ox.un4

MAPAIEEEHVDSLSSNGQKLCIKNFIWSDSEWPQINYDHFDDRDDIPLISLQKDQNCEKVCRDMVAASNKWGFFKLVDHGVPSEIVENMKARLNEMFDLPMEEKLKGARSATMPLGYCTTNPDYGKNLPWAEILQLLQSPQQVLGFAKKVFGDQHQPFSEAMVEYMEALDKLGMTIMEMLANGLGLQSDFFTKNFEEKDSTMIRINKYPPCPLPEKCLGVGSHTDPHTLTILLADNVGGLQVLKDDNQWVGIRPIPNSFVINIGDTLEAWTNGRLKSVIHRAVVNKEKSRLSIAYFLSPALSTIIDSPPELLEDPNTDPPINRKYVPFTWADFRKELLIQKRVIGKTALNRYLISP

>CsGA2ox.un6

MVVSSPTPFRTKKARAVGIPIIDLSLERSVLSEKIVQACQDYGFFKVVNHGVPMEIIARMEKEGSDFFAKPASEKQQQAGDLPSPFGYGCKNIGFNGDVGELEYLLLHTNHLSISDISKAISSINPTKFSCAVNDYIQAVRSLTCEMLDLVAEGLFVPDKSVFSRLIGDVHSDSCFRLNHYPPMENSREWDPSPKLNANTRMGFGEHSDPQILTILRSNDVEGLQICLHDGLWVPVTPDPTEFCVFVGYDKWKVCERKTQSNSSKLSETQNVNDIFWGSTP

>CsGA2ox.un8

MVENWCPSNQAMVKIDKIPVHQSQSSSMDSSEPPFQEAYKTLFLNYTEAESNHANELIIVKECELPVIDLSRLELGESEREECKREIAKASQEWGFFQVVNHGISTEILERMRCEQVKVFKRPFREKIREGYMNFSAGSYRWGTPWATCLRQISWSEAFHVPLDDICGLGGLTTLSSTMEQFATTVSDLAQKLAEILAEKMGHKATFFKENCVPSTCYLRMNRYPPCPISSEIFGLIPHTDSDFLTVLHQDQIGGLQLVKDGKWIAVKPNPDALIINIGDLFQVWSNGVYRSVEHRVVANKKMERFSTAYFFCPSYDTLMQSCVEPSIYRRFSFREFRQQVQEDVKRFGHKIGLPRFLV

>CsGA20ox10

MNSLQSWPEPIIRVQSLSDSGLTFIPDQYVKPLADRPSLTEPPPPPAEINIPVIDLSQLFSPDRSIRSATARVISRACSEWGFFQVVNHGVSHELMKRIREVWREFFELPSEEKQAYANSPATYEGYGSRLGVEKGMKLDWSDYFFLHYLPESLRDQNKWPSLPLSCRELVAEYGNELIKLCGKLMKVFSVNLGLEEDYLGKAFGGEEVAACMRVNFYPKCPQPDLTLGLSSHSDPGGMTLLHSDDHVAGLQVRRGDDWVTVKPVPNAFIVNIGDQIQILSNATYKSVEHRVIVNSAKERLSLAFFYNPNGNILIEPASELVTEDSPALYPAMTFNKYRLFIRTKGPRGKSQVEALKSPH

> CsGA2ox.un

MKEREERKRRYNEMLKEREERIKLANEMLKEREEGQKARKETNTTVEHLNNVVAQTVTTLGNLSWGIPTFAFAIATERSPSSLSINWVEGFNVPLSQLSVLQADGDPILDTFRHLLDEYGRHQSRVATTIFKAMAENLNLLGQEKCKSYISPSTSFIRVYRYPRCSESNHNNRASAWGMDAHTDSSVISILNHHDQVGGLEVYKDHKWLDIKPIPNTLVVNLGDMTQAMSDDEYKSVKHRVKANKYDDRISIGYFVFPAQNGVIQSSKYKPFTYSDFQAQVQKDLMTMGFKVGLDRFKLNQAS

>CsGA2ox4.2

MEKQKQQRRTSTFFDFCKAEKGILLCTDVAARGLDIPAVVDDKYQYSTEDKDNQVHGWICFDPPVGFWQITPSNEFRTGGPFKQDLTSHVNPTTLAVEQKRKEALSRTGEESQSALESSLQDVVSCYSFMDLWPCSSKDMDHLARQEVMTNGRFKSVRHRVIANSLKSRVSMIYFGGPPLSEKIAPLPSLMEGEDSLYKEFTWFEYKKSAYKSRLADNRLGLFEKIAAS

>CsGA3ox5.5

MENLAVKHRVMMSGEKERYSCGFFTIPKDGVGIKVPQELVDKNHPLLYLPFIYSEYLQKHLTDLSFNDMNVMLELNLLLLRMIMESYGVGEYYKSKAFGDNTGNNFRAMKYKVPKSKNEEGIGLGPHVDKTILTFLCDNSVRGLEALTQDGIWISLHIPKGALLVMVGDALEVLSNGRLQAAKHRVIMSGEKERYSCAFYTIPKDGVGIEVPQELVDEDHPLLYRPFVYSEYLEKHLTNTNFDALQVHAGI

>CsGA3ox8.2

MGEAAEMIPSISLTNEVLSLDRHSEEWKAMTGKVREACEAYGCFMLDYEKIPLELQQILFKTTAEMFDLPVETKLQYKKPTPKDGGYVLSNPFIPLYESFGVYGEEEVRAFERLMWPQGNPQFCEVINSITAKVHELNLLLLRMIFESFGIEEYYKEKALDENIIGYLRSMKYRLPKEDEDPLGLAIHVDKTIITILCQNSVQGLEVLTKEGKWIKLSIPKGTLVVIVGDGLQAWTNGRVEAVQHRVVMSGDKERLSCGFFSIPKDGVSVEIHPQLVDEEHPLRYRSFVYSDYLAKYLANTRLDALELFAGIC

>CsGA2ox3.1

MAVSPESSADQNPVDFRAPPPSPIASGRRSSFKNDDILTDFLEHSLRVPDLILPDRFFPRQKSIQNPPKIDFQSLNSPENDDSVAEMLDAIARFGFFQIVNHGITREQIASVSMSGAGIFRLSPEKKAVVSRSPEKPYGFEEFHGEVESEMSEEFVWCRDHGLKLEMEGVWPLGYSKFSENMEMLLVEIEKVGGRVVLAMEEHNARKSQCGNGIIRGQDFDGSVCCFYKHSRNVSGDQWVGSLKCDVIKMLIRGSDYSHALCMHVCEGSSEFHVYSKKGWVSFCPDTDAIVITIGDQIQAWSGGLYKQVIGRPIFKGENEECISMAFLYSPSKNIKSFKTNEGKTISLGQQAIVAILFTLVYHFLVYIYKL

>CsGA2ox7.2

MAHVQRTSFDERDVEQVFGVYYAELDPIWRCLIEVMTNGRFKSVRHRVIANSLKSRVSMIYFGGPPLSEKIAPLPSLMEGEDSLYKEFTWFECKKSAYKSRLADNRLGLFEKIAAS

>AtGA20ox1

MAVSFVTTSPEEEDKPKLGLGNIQTPLIFNPSMLNLQANIPNQFIWPDDEKPSINVLELDVPLIDLQNLLSDPSSTLDASRLISEACKKHGFFLVVNHGISEELISDAHEYTSRFFDMPLSEKQRVLRKSGESVGYASSFTGRFSTKLPWKETLSFRFCDDMSRSKSVQDYFCDALGHGFQPFGKVYQEYCEAMSSLSLKIMELLGLSLGVKRDYFREFFEENDSIMRLNYYPPCIKPDLTLGTGPHCDPTSLTILHQDHVNGLQVFVENQWRSIRPNPKAFVVNIGDTFMALSNDRYKSCLHRAVVNSESERKSLAFFLCPKKDRVVTPPRELLDSITSRRYPDFTWSMFLEFTQKHYRADMNTLQAFSDWLTKPI

>AtGA20ox2

MAILCTTTSPAEKEHEPKQDLEKDQTSPLIFNPSLLNLQSQIPNQFIWPDEEKPSIDIPELNVPFIDLSSQDSTLEAPRVIAEACTKHGFFLVVNHGVSESLIADAHRLMESFFDMPLAGKQKAQRKPGESCGYASSFTGRFSTKLPWKETLSFQFSNDNSGSRTVQDYFSDTLGQEFEQFGKVYQDYCEAMSSLSLKIMELLGLSLGVNRDYFRGFFEENDSIMRLNHYPPCQTPDLTLGTGPHCDPSSLTILHQDHVNGLQVFVDNQWQSIRPNPKAFVVNIGDTFMALSNGIFKSCLHRAVVNRESARKSMAFFLCPKKDKVVKPPSDILEKMKTRKYPDFTWSMFLEFTQKHYRADVNTLDSFSNWVITNNNPI

>AtGA20ox3

MATECIATVPQIFSENKTKEDSSIFDAKLLNQHSHHIPQQFVWPDHEKPSTDVQPLQVPLIDLAGFLSGDSCLASEATRLVSKAATKHGFFLITNHGVDESLLSRAYLHMDSFFKAPACEKQKAQRKWGESSGYASSFVGRFSSKLPWKETLSFKFSPEEKIHSQTVKDFVSKKMGDGYEDFGKVYQEYAEAMNTLSLKIMELLGMSLGVERRYFKEFFEDSDSIFRLNYYPQCKQPELALGTGPHCDPTSLTILHQDQVGGLQVFVDNKWQSIPPNPHAFVVNIGDTFMALTNGRYKSCLHRAVVNSERERKTFAFFLCPKGEKVVKPPEELVNGVKSGERKYPDFTWSMFLEFTQKHYRADMNTLDEFSIWLKNRRSF

>AtGA20ox4

MECIIKLPQRFNKNKSKKNPLRIFDSTVLNHQPDHIPQEFVWPDHEKPSKNVPILQVPVIDLAGFLSNDPLLVSEAERLVSEAAKKHGFFLVTNHGVDERLLSTAHKLMDTFFKSPNYEKLKAQRKVGETTGYASSFVGRFKENLPWKETLSFSFSPTEKSENYSQTVKNYISKTMGDGYKDFGSVYQEYAETMSNLSLKIMELLGMSLGIKREHFREFFEDNESIFRLNYYPKCKQPDLVLGTGPHCDPTSLTILQQDQVSGLQVFVDNQWQSIPPIPQALVVNIGDTLMALTNGIYKSCLHRAVVNGETTRKTLAFFLCPKVDKVVKPPSELEGERAYPDFTWSMFLEFTMKHYRADMNTLEEFTNWLKNKGSF

>AtGA20ox5

MCIYASRQTVCPYLTPFKVKRPKSREMNSSDVNFSLLQSQPNVPAEFFWPEKDVAPSEGDLDLPIIDLSGFLNGNEAETQLAAKAVKKACMAHGTFLVVNHGFKSGLAEKALEISSLFFGLSKDEKLRAYRIPGNISGYTAGHSQRFSSNLPWNETLTLAFKKGPPHVVEDFLTSRLGNHRQEIGQVFQEFCDAMNGLVMDLMELLGISMGLKDRTYYRRFFEDGSGIFRCNYYPPCKQPEKALGVGPHNDPTAITVLLQDDVVGLEVFAAGSWQTVRPRPGALVVNVGDTFMALSNGNYRSCYHRAVVNKEKVRRSLVFFSCPREDKIIVPPPELVEGEEASRKYPDFTWAQLQKFTQSGYRVDNTTLHNFSSWLVSNSDKKST

>AtGA3ox1

MPAMLTDVFRGHPIHLPHSHIPDFTSLRELPDSYKWTPKDDLLFSAAPSPPATGENIPLIDLDHPDATNQIGHACRTWGAFQISNHGVPLGLLQDIEFLTGSLFGLPVQRKLKSARSETGVSGYGVARIASFFNKQMWSEGFTITGSPLNDFRKLWPQHHLNYCDIVEEYEEHMKKLASKLMWLALNSLGVSEEDIEWASLSSDLNWAQAALQLNHYPVCPEPDRAMGLAAHTDSTLLTILYQNNTAGLQVFRDDLGWVTVPPFPGSLVVNVGDLFHILSNGLFKSVLHRARVNQTRARLSVAFLWGPQSDIKISPVPKLVSPVESPLYQSVTWKEYLRTKATHFNKALSMIRNHREE

>AtGA3ox2

MSSTLSDVFRSHPIHIPLSNPPDFKSLPDSYTWTPKDDLLFSASASDETLPLIDLSDIHVATLVGHACTTWGAFQITNHGVPSRLLDDIEFLTGSLFRLPVQRKLKAARSENGVSGYGVARIASFFNKKMWSEGFTVIGSPLHDFRKLWPSHHLKYCEIIEEYEEHMQKLAAKLMWFALGSLGVEEKDIQWAGPNSDFQGTQAVIQLNHYPKCPEPDRAMGLAAHTDSTLMTILYQNNTAGLQVFRDDVGWVTAPPVPGSLVVNVGDLLHILTNGIFPSVLHRARVNHVRSRFSMAYLWGPPSDIMISPLPKLVDPLQSPLYPSLTWKQYLATKATHFNQSLSIIRN

>AtGA3ox3

MSSVTQLFKNNPVNRDRIIPLDFTNTKTLPDSHVWSKPEPETTSGPIPVISLSNPEEHGLLRQACEEWGVFHITDHGVSHSLLHNVDCQMKRLFSLPMHRKILAVRSPDESTGYGVVRISMFYDKLMWSEGFSVMGSSLRRHATLLWPDDHAEFCNVMEEYQKAMDDLSHRLISMLMGSLGLTHEDLGWLVPDKTGSGTDSIQSFLQLNSYPVCPDPHLAMGLAPHTDSSLLTILYQGNIPGLEIESPQEEGSRWIGVEPIEGSLVVIMGDLSHIISNGQFRSTMHRAVVNKTHHRVSAAYFAGPPKNLQIGPLTSDKNHPPIYRRLIWEEYLAAKATHFNKALTLFRC

>AtGA3ox4

MPSLAEEICIGNLGSLQTLPESFTWKLTAADSLLRPSSAVSFDAVEESIPVIDLSNPDVTTLIGDASKTWGAFQIANHGISQKLLDDIESLSKTLFDMPSERKLEAASSDKGVSGYGEPRISPFFEKKMWSEGFTIADDSYRNHFNTLWPHDHTKYCGIIQEYVDEMEKLASRLLYCILGSLGVTVEDIEWAHKLEKSGSKVGRGAIRLNHYPVCPEPERAMGLAAHTDSTILTILHQSNTGGLQVFREESGWVTVEPAPGVLVVNIGDLFHILSNGKIPSVVHRAKVNHTRSRISIAYLWGGPAGDVQIAPISKLTGPAEPSLYRSITWKEYLQIKYEVFDKAMDAIRVVNPTN

>AtGA2ox1

MAVLSKPVAIPKSGFSLIPVIDMSDPESKHALVKACEDFGFFKVINHGVSAELVSVLEHETVDFFSLPKSEKTQVAGYPFGYGNSKIGRNGDVGWVEYLLMNANHDSGSGPLFPSLLKSPGTFRNALEEYTTSVRKMTFDVLEKITDGLGIKPRNTLSKLVSDQNTDSILRLNHYPPCPLSNKKTNGGKNVIGFGEHTDPQIISVLRSNNTSGLQINLNDGSWISVPPDHTSFFFNVGDSLQVMTNGRFKSVRHRVLANCKKSRVSMIYFAGPSLTQRIAPLTCLIDNEDERLYEEFTWSEYKNSTYNSRLSDNRLQQFERKTIKNLLN

>AtGA2ox2

MVVLPQPVTLDNHISLIPTYKPVPVLTSHSIPVVNLADPEAKTRIVKACEEFGFFKVVNHGVRPELMTRLEQEAIGFFGLPQSLKNRAGPPEPYGYGNKRIGPNGDVGWIEYLLLNANPQLSSPKTSAVFRQTPQIFRESVEEYMKEIKEVSYKVLEMVAEELGIEPRDTLSKMLRDEKSDSCLRLNHYPAAEEEAEKMVKVGFGEHTDPQIISVLRSNNTAGLQICVKDGSWVAVPPDHSSFFINVGDALQVMTNGRFKSVKHRVLADTRRSRISMIYFGGPPLSQKIAPLPCLVPEQDDWLYKEFTWSQYKSSAYKSKLGDYRLGLFEKQPLLNHKTLV

>AtGA2ox3

MVIVLQPASFDSNLYVNPKCKPRPVLIPVIDLTDSDAKTQIVKACEEFGFFKVINHGVRPDLLTQLEQEAINFFALHHSLKDKAGPPDPFGYGTKRIGPNGDLGWLEYILLNANLCLESHKTTAIFRHTPAIFREAVEEYIKEMKRMSSKFLEMVEEELKIEPKEKLSRLVKVKESDSCLRMNHYPEKEETPVKEEIGFGEHTDPQLISLLRSNDTEGLQICVKDGTWVDVTPDHSSFFVLVGDTLQVMTNGRFKSVKHRVVTNTKRSRISMIYFAGPPLSEKIAPLSCLVPKQDDCLYNEFTWSQYKLSAYKTKLGDYRLGLFEKRPPFSLSNV

>AtGA2ox4

MVKGSQKIVAVDQDIPIIDMSQERSQVSMQIVKACESLGFFKVINHGVDQTTISRMEQESINFFAKPAHEKKSVRPVNQPFRYGFRDIGLNGDSGEVEYLLFHTNDPAFRSQLSFSSAVNCYIEAVKQLAREILDLTAEGLHVPPHSFSRLISSVDSDSVLRVNHYPPSDQFFGEANLSDQSVSLTRVGFGEHTDPQILTVLRSNGVGGLQVSNSDGMWVSVSPDPSAFCVNVGDLLQVMTNGRFISVRHRALTYGEESRLSTAYFAGPPLQAKIGPLSAMVMTMNQPRLYQTFTWGEYKKRAYSLRLEDSRLDMFRTCKD

>AtGA2ox6

MVLPSSTPLQTTGKKTISSPEYNFPVIDFSLNDRSKLSEKIVKACEVNGFFKVINHGVKPEIIKRFEHEGEEFFNKPESDKLRAGPASPFGYGCKNIGFNGDLGELEYLLLHANPTAVADKSETISHDDPFKFSSATNDYIRTVRDLACEIIDLTIENLWGQKSSEVSELIRDVRSDSILRLNHYPPAPYALSGVGQIGFGEHSDPQILTVLRSNDVDGLEICSRDGLWIPIPSDPTCFFVLVGDCLQALTNGRFTSVRHRVLANTAKKPRMSAMYFAAPPLEAKISPLPKMVSPENPRRYNSFTWGDYKKATYSLRLDVPRLEFFKTL

>AtGA2ox7

MASQPPFKTNFCSIFGSSFPNSTSESNTNTSTIQTSGIKLPVIDLSHLTSGEEVKRKRCVKQMVAAAKEWGFFQIVNHGIPKDVFEMMLLEEKKLFDQPFSVKVRERFSDLSKNSYRWGNPSATSPAQYSVSEAFHIILSEVSRISDDRNNLRTIVETYVQEIARVAQMICEILGKQVNVSSEYFENIFELENSFLRLNKYHPSVFGSEVFGLVPHTDTSFLTILSQDQIGGLELENNGQWISVKPCLEALTVNIGDMFQALSNGVYQSVRHRVISPANIERMSIAFFVCPYLETEIDCFGYPKKYRRFSFREYKEQSEHDVKETGDKVGLSRFLI

>AtGA2ox8

MDPPFNEIYNNLLYNQITKKDNDVSEIPFSFSVTAVVEEVELPVIDVSRLIDGAEEEREKCKEAIARASREWGFFQVINHGISMDVLEKMRQEQIRVFREPFDKKSKSEKFSAGSYRWGTPSATSIRQLSWSEAFHVPMTDISDNKDFTTLSSTMEKFASESEALAYMLAEVLAEKSGQNSSFFKENCVRNTCYLRMNRYPPCPKPSEVYGLMPHTDSDFLTILYQDQVGGLQLIKDNRWIAVKPNPKALIINIGDLFQAWSNGMYKSVEHRVMTNPKVERFSTAYFMCPSYDAVIECSSDRPAYRNFSFREFRQQVQEDVKKFGFKVGLPRFLNHVY
