# Supplementary material for: Genome-wide identification and integrated analysis of gibberellin oxidase genes associated with branch angle and internode elongation in tea plants (Camellia sinensis)
Source: Front Plant Sci. 2026 Sep 3;17:1940210. doi: 10.3389/fpls.2026.1940210 (PMC13582540; doi:10.3389/fpls.2026.1940210)
Supplement: Supplementary file 1 [file DataSheet1.zip › Supplementary Files/Table S1.docx]

**Table S1. Genomic information and physicochemical properties of 33 putative *CsGAox* family genes in tea cultivar LJ43.**

| **Sequence ID** | **Gene Name** | **CDS length (bp)** | **AA**  **number** | **MW (kDa)** | **pI** | **Instability Index** | **GRAVY** |
| --- | --- | --- | --- | --- | --- | --- | --- |
| Cha01g015700 | *CsGA2ox1.2* | 912 | 303 | 33.66 | 5.73 | 47.55 | -0.12 |
| Cha01g005450 | *CsGA2ox1.1* | 1149 | 382 | 42.04 | 4.76 | 45.83 | -0.42 |
| Cha04g014660 | *CsGA2ox4.1* | 1062 | 353 | 40.04 | 6.99 | 36.14 | -0.48 |
| Cha04g018570 | *CsGA20ox4.3* | 954 | 317 | 36.54 | 6.84 | 47.94 | -0.62 |
| Cha05g002010 | *CsGA2ox5.1* | 1035 | 344 | 38.70 | 5.73 | 47.33 | -0.21 |
| Cha05g011540 | *CsGA2ox5.2* | 951 | 316 | 34.88 | 5.29 | 44.31 | -0.14 |
| Cha05g011580 | *CsGA2ox5.3* | 873 | 290 | 32.87 | 6 | 40.11 | -0.25 |
| Cha05g011590 | *CsGA2ox5.4* | 999 | 332 | 37.31 | 7.61 | 33.16 | -0.23 |
| Cha06g008080 | *CsGA3ox6* | 1032 | 343 | 38.44 | 4.93 | 50.78 | -0.26 |
| Cha07g008300 | *CsGA2ox7.1* | 1005 | 334 | 37.63 | 5.47 | 40.04 | -0.22 |
| Cha07g013670 | *CsGA2ox7.3* | 1239 | 412 | 47.41 | 5.73 | 48.86 | -0.36 |
| Cha08g016320 | *CsGA2ox8.4* | 1143 | 380 | 43.15 | 5.97 | 37.32 | -0.31 |
| Cha08g009400 | *CsGA3ox8.3* | 945 | 314 | 35.67 | 4.81 | 38.11 | -0.13 |
| Cha08g004600 | *CsGA2ox8.1* | 1134 | 377 | 41.42 | 5.02 | 53.89 | -0.31 |
| Cha09g017700 | *CsGA2ox9* | 570 | 189 | 21.30 | 6.44 | 40.96 | -0.22 |
| Cha11g000330 | *CsGA2ox11* | 1029 | 342 | 38.24 | 8.33 | 44.78 | -0.15 |
| Cha14g001160 | *CsGA3ox14* | 1038 | 345 | 38.74 | 5.69 | 43.27 | -0.13 |
| Cha15g009060 | *CsGA3ox15* | 1326 | 441 | 49.11 | 7.66 | 41.8 | -0.25 |
| Cha04g021780 | *CsGA20ox4.4* | 1080 | 359 | 40.45 | 6.11 | 52.88 | -0.28 |
| Cha03g006460 | *CsGA3ox3.2* | 492 | 163 | 17.95 | 6.21 | 39.44 | -0.28 |
| ChaUn1649.3 | *CsGA2ox.un1* | 858 | 285 | 31.91 | 5.88 | 35.04 | -0.14 |
| Cha03g006450 | *CsGA3ox3.3* | 861 | 286 | 31.90 | 9.92 | 29.11 | -0.24 |
| ChaUn6953.7 | *CsGA20ox.un3* | 969 | 323 | 36.61 | 8.22 | 38.72 | -0.34 |
| ChaUn6565.4 | *CsGA2ox.un4* | 1071 | 356 | 40.34 | 5.3 | 41.21 | -0.33 |
| ChaUn13888.1 | *CsGA2ox.un6* | 846 | 281 | 31.31 | 5.4 | 40.03 | -0.25 |
| ChaUn8759.2 | *CsGA2ox.un8* | 1080 | 359 | 41.33 | 6.18 | 51.16 | -0.32 |
| Cha10g004160 | *CsGA20ox10* | 1083 | 360 | 40.50 | 5.96 | 56.25 | -0.31 |
| ChaUn12153.4 | *CsGA2ox.un* | 912 | 303 | 34.76 | 8.65 | 29.78 | -0.63 |
| Cha04g016560 | *CsGA2ox4.2* | 690 | 229 | 26.09 | 6.9 | 42.57 | -0.55 |
| Cha05g016340 | *CsGA3ox5.5* | 756 | 251 | 28.46 | 6 | 25.66 | -0.19 |
| Cha08g009430 | *CsGA3ox8.2* | 942 | 313 | 35.70 | 5.07 | 27.45 | -0.15 |
| Cha03g005890 | *CsGA2ox3.1* | 1116 | 371 | 41.65 | 5.64 | 45.4 | -0.23 |
| Cha07g008840 | *CsGA2ox7.2* | 351 | 116 | 13.40 | 8.58 | 32.67 | -0.28 |

Abbreviation: AA=amino acids, MS = molecular mass, pI= isoelectric point, and GRAVY= grand average of hydropathy.
