# Supplementary material for: Genome-wide identification and integrated analysis of gibberellin oxidase genes associated with branch angle and internode elongation in tea plants (Camellia sinensis)
Source: Front Plant Sci. 2026 Sep 3;17:1940210. doi: 10.3389/fpls.2026.1940210 (PMC13582540; doi:10.3389/fpls.2026.1940210)
Supplement: Supplementary file 1 [file DataSheet1.zip › Supplementary Files/Supplementary Figure and Table Legends.docx]

**Supplementary Figure Legends**

**Figure S1. Chromosomal distribution of *CsGAox* genes in the LJ43 genome.** The chromosomal locations of 27 *CsGAox* genes on 12 chromosomes are shown. Chromosome numbers are indicated on the left, and gene positions are shown according to their physical locations.

**Figure S2. Branch angles of three tea cultivars with different plant architectures.** Branch angles of the draped cultivar DT and the two upright cultivars UT1 and UT2. Data are presented as means ± SE of three independent biological replicates. ** indicates a significant difference at *P* < 0.01.

**Supplementary Table Legends**

**Table S1. Genomic information and physicochemical properties of 33 putative CsGAox family genes in tea cultivar LJ43.**

**Table S2. Transcript sequences of *CsGAox* genes in LJ43 and protein sequences of CsGAox and Arabidopsis AtGAox genes used in this study.**

**Table S3. Identification and classification summary of *GAox* genes across eight *Camellia* genomes.**

**Table S4. Subcellular localization predictions of 33 CsGAox proteins in LJ43.**

**Table S5. Putative cis-acting elements in the promoter regions of *CsGAox* genes in LJ43.**

**Table S6. Duplication types of *GAox* genes across eight *Camellia* genomes.**

**Table S7. Collinear *GAox* gene pairs within individual *Camellia* genomes.**

**Table S8. Ka, Ks, and Ka/Ks values of duplicated *GAox* gene pairs in six *Camellia* genomes.**

**Table S9. Syntenic gene pairs involving LJ43 *CsGAox* genes and their counterparts in six other *Camellia* genomes.**

**Table S10. Correlation analysis among branch angle, GA contents, and *CsGAox* gene expression.**

**Table S11. Correlation analysis between internode length and *CsGAox* gene expression.**
